# Management of intra-abdominal infections: recommendations by the WSES 2016 consensus conference

**DOI:** 10.1186/s13017-017-0132-7

**Published:** 2017-05-04

**Authors:** Massimo Sartelli, Fausto Catena, Fikri M. Abu-Zidan, Luca Ansaloni, Walter L. Biffl, Marja A. Boermeester, Marco Ceresoli, Osvaldo Chiara, Federico Coccolini, Jan J. De Waele, Salomone Di Saverio, Christian Eckmann, Gustavo P. Fraga, Maddalena Giannella, Massimo Girardis, Ewen A. Griffiths, Jeffry Kashuk, Andrew W. Kirkpatrick, Vladimir Khokha, Yoram Kluger, Francesco M. Labricciosa, Ari Leppaniemi, Ronald V. Maier, Addison K. May, Mark Malangoni, Ignacio Martin-Loeches, John Mazuski, Philippe Montravers, Andrew Peitzman, Bruno M. Pereira, Tarcisio Reis, Boris Sakakushev, Gabriele Sganga, Kjetil Soreide, Michael Sugrue, Jan Ulrych, Jean-Louis Vincent, Pierluigi Viale, Ernest E. Moore

**Affiliations:** 1 Department of Surgery, Macerata Hospital, Macerata, Italy; 2 Department of Emergency Surgery, Maggiore Hospital, Parma, Italy; 3 Department of Surgery, College of Medicine and Health Sciences, UAE University, Al-Ain, United Arab Emirates; 4 General Surgery Department, Papa Giovanni XXIII Hospital, Bergamo, Italy; 5 Acute Care Surgery, The Queen’s Medical Center, Honolulu, HI USA; 6 Department of Surgery, Academic Medical Centre, Amsterdam, Netherlands; 7 Emergency Department, Trauma Center, Niguarda Hospital, Milan, Italy; 8 Department of Critical Care Medicine, Ghent University Hospital, Ghent, Belgium; 9 Department of Surgery, Maggiore Hospital, Bologna, Italy; 10 Department of General, Visceral, and Thoracic Surgery, Klinikum Peine, Academic Hospital of Medical University Hannover, Hannover, Germany; 11 Division of Trauma Surgery, Department of Surgery, School of Medical Sciences, University of Campinas, Campinas, Brazil; 12 Infectious Diseases Unit, Department of Medical and Surgical Sciences, Sant’Orsola Hospital, University of Bologna, Bologna, Italy; 13 Intensive Care Unit, University of Modena, Modena, Italy; 14 General and Upper GI Surgery, Queen Elizabeth Hospital, Birmingham, UK; 15 Department of Surgery, Assia Medical Group, Tel Aviv University Sackler School of Medicine, Tel Aviv, Israel; 16 Departments of Surgery, Critical Care Medicine, and the Regional Trauma Service, Foothills Medical Centre, Calgary, AB Canada; 17 Department of Emergency Surgery, Mozyr City Hospital, Mozyr, Belarus; 18 Department of General Surgery, Division of Surgery, Rambam Health Care Campus, Haifa, Israel; 19 Department of Biomedical Sciences and Public Health, Unit of Hygiene, Preventive Medicine and Public Health, UNIVPM, Ancona, Italy; 20 Abdominal Center, University Hospital Meilahti, Helsinki, Finland; 21 Department of Surgery, University of Washington, Seattle, WA USA; 22 Departments of Surgery and Anesthesiology, Division of Trauma and Surgical Critical Care, Vanderbilt University Medical Center, Nashville, TN USA; 23 American Board of Surgery, Philadelphia, PA USA; 24 Multidisciplinary Intensive Care Research Organization (MICRO), Wellcome Trust-HRB Clinical Research, Department of Clinical Medicine, Trinity Centre for Health Sciences, St James’s University Hospital, Dublin, Ireland; 25 Department of Surgery, School of Medicine, Washington University in Saint Louis, St. Louis, MO USA; 26 Département d’Anesthésie-Réanimation, CHU Bichat Claude-Bernard-HUPNVS, Assistance Publique-Hôpitaux de Paris, University Denis Diderot, Paris, France; 27 Department of Surgery, UPMC, University of Pittsburgh School of Medicine, Pittsburgh, PA USA; 28 Emergency post-operative Department, Otavio De Freitas Hospital and Osvaldo Cruz Hospital Recife, Recife, Brazil; 29 General Surgery Department, Medical University, University Hospital St George, Plovdiv, Bulgaria; 30 Department of Surgery, Catholic University of Sacred Heart, Rome, Italy; 31 Department of Gastrointestinal Surgery, Stavanger University Hospital, Stavanger, Department of Clinical Medicine, University of Bergen, Bergen, Norway; 32 Letterkenny University Hospital and Donegal Clinical Research Academy, Letterkenny, Ireland; 33 1st Department of Surgery, Department of Abdominal, Thoracic Surgery and Traumatology, General University Hospital, Praha, Czech Republic; 34 Department of Intensive Care, Erasme Hospital, Université libre de Bruxelles, Brussels, Belgium; 35 Department of Surgery, University of Colorado, Denver, CO USA

**Keywords:** Intra-abdominal infections, Sepsis, Peritonitis, Antibiotics

## Abstract

This paper reports on the consensus conference on the management of intra-abdominal infections (IAIs) which was held on July 23, 2016, in Dublin, Ireland, as a part of the annual World Society of Emergency Surgery (WSES) meeting. This document covers all aspects of the management of IAIs. The Grading of Recommendations Assessment, Development and Evaluation recommendation is used, and this document represents the executive summary of the consensus conference findings.

## Background

Intra-abdominal infections (IAIs) are an important cause of morbidity and mortality [1]. Early clinical diagnosis, adequate source control to stop ongoing contamination, appropriate antimicrobial therapy dictated by patient and infection risk factors, and prompt resuscitation in critically ill patients are the cornerstones in the management of IAIs. However, several critical controversies can be debated in the management of these patients. Application of management principles to the individual patient is crucial to optimize outcome. In order to clarify these major controversies in the management of IAI, many of the world’s leading experts met in Dublin, Ireland, on July 23, 2016, for a specialist multidisciplinary consensus conference under the auspices of the World Society of Emergency Surgery (WSES) and with the support of the World Society of Abdominal Compartment Syndrome (WSACS). This document represents the executive summary of this consensus conference.

## Methodology

In constituting the expert panel, the WSES committee invited many of the world’s leading experts in the management of IAIs. It was a multidisciplinary expert panel including general and specialist surgeons, emergency and acute care surgeons, infectious disease specialists, and intensivists. The expert panel reviewed the scientific evidence and composed statements which addressed a set of predefined questions.

The statements were formulated and graded according to the Grading of Recommendations Assessment, Development and Evaluation (GRADE) hierarchy of evidence from Guyatt and colleagues [1], summarized in Table 1.

**Table 1:** Grading of Recommendations Assessment, Development and Evaluation (GRADE) hierarchy of evidence from Guyatt and colleagues [1]

Grade of recommendation	Clarity of risk/benefit	Quality of supporting evidence	Implications
1A			
Strong recommendation, high-quality evidence	Benefits clearly outweigh risk and burdens, or vice versa	RCTs without important limitations or overwhelming evidence from observational studies	Strong recommendation, applies to most patients in most circumstances without reservation
1B			
Strong recommendation, moderate-quality evidence	Benefits clearly outweigh risk and burdens, or vice versa	RCTs with important limitations (inconsistent results, methodological flaws, indirect analyses or imprecise conclusions) or exceptionally strong evidence from observational studies	Strong recommendation, applies to most patients in most circumstances without reservation
1C			
Strong recommendation, low-quality or very low-quality evidence	Benefits clearly outweigh risk and burdens, or vice versa	Observational studies or case series	Strong recommendation but subject to change when higher quality evidence becomes available
2A			
Weak recommendation, high-quality evidence	Benefits closely balanced with risks and burden	RCTs without important limitations or overwhelming evidence from observational studies	Weak recommendation, best action may differ depending on the patient, treatment circumstances, or social values
2B			
Weak recommendation, moderate-quality evidence	Benefits closely balanced with risks and burden	RCTs with important limitations (inconsistent results, methodological flaws, indirect or imprecise) or exceptionally strong evidence from observational studies	Weak recommendation, best action may differ depending on the patient, treatment circumstances, or social values
2C			
Weak recommendation, Low-quality or very low-quality evidence	Uncertainty in the estimates of benefits, risks, and burden; benefits, risk, and burden may be closely balanced	Observational studies or case series	Very weak recommendation; alternative treatments may be equally reasonable and merit consideration

During the WSES annual conference which was held in Dublin, the statements were debated and approved by the committee jury panel. This document represents the executive summary of the final recommendations approved by the consensus conference. The document was reviewed and approved by both the expert and jury panels.

## What is the most appropriate classification for intra-abdominal infections?

**Statement 1**

**The term “intra-abdominal infections” describes a wide heterogeneity of clinical conditions. The anatomical extent of infection, the presumed pathogens involved, risk factors for major resistance patterns, and the patient's clinical condition should be assessed independently so as to classify patients (Recommendation 1 C).**

A complete classification that includes the origin of source of infection, the anatomical extent of infection, the presumed pathogens involved and risk factors for major resistance patterns, and the patient’s clinical condition does not exist.

A simple and universally accepted classification divides IAIs into complicated and uncomplicated [2]. In the event of uncomplicated IAIs, the infection only involves a single organ and does not extend to the peritoneum. When the focus of infection is controlled by surgical excision, post-operative antibiotic therapy is not necessary [3–5]. In the event of complicated IAIs (cIAIs), the infectious process proceeds beyond the organ into the peritoneum, causing either localized or diffuse peritonitis.

Treatment of patients with cIAIs involves generally both source control, antibiotic therapy, and potentially novel therapies targeted at modulating or ameliorating the host inflammatory response. Antibiotics can prevent local and hematogenous spread and can reduce late complications [6].

This classification has been questioned by some authors because it often has incited confusion by mixing elements of anatomical barrier disruption and severity of disease expression [7].

Peritonitis is an inflammation of the peritoneum. Depending on the underlying pathology, it can be infectious or sterile. Infectious peritonitis is classified into primary peritonitis, secondary peritonitis, and tertiary peritonitis [2]. Primary peritonitis is a diffuse bacterial infection (usually caused by a single organism) without loss of integrity of the gastrointestinal tract, typically seen in cirrhotic patients with ascites or in patients with a peritoneal dialysis catheter. It has a low incidence on surgical wards and is usually managed without any surgical intervention. Secondary peritonitis, the most common form of peritonitis, is an acute peritoneal infection resulting from loss of integrity of the gastrointestinal tract. Tertiary peritonitis is a recurrent infection of the peritoneal cavity that occurs >48 h after apparently successful and adequate surgical source control of secondary peritonitis. It is more common among critically ill or immunocompromised patients and may be often associated with multidrug-resistant organisms (MDROs). It is typically associated with high morbidity and mortality. Tertiary peritonitis has been accepted as a distinct entity. However, it represents an evolution and complication of secondary peritonitis [8–10]. The term “ongoing peritonitis” [11] or “persistent peritonitis” [12] may better indicate that it is not a different disease to secondary peritonitis, but rather represents secondary peritonitis lasting longer and harboring other (selected and more resistant) pathogens.

The term “healthcare-associated infection” (HCAI) is a new term for infections acquired during the course of receiving healthcare. It includes not only hospital-acquired infections but also infections in patients living in nursing facilities, having recent hospitalization within 90 days, and using aggressive medical therapies (intravenous therapy, wound dressing) at home and invasive therapies (hemodialysis, chemotherapy, radiotherapy) in outpatient clinics within 30 days of the index infection [13]. In the setting of IAIs, there is relatively little data regarding the concept of healthcare-associated infections as opposed to hospital-acquired or nosocomial infections.

Differentiating community-acquired intra-abdominal infections (CA-IAIs) from hospital-acquired intra-abdominal infections (HA-IAIs) is useful to define the presumed resistance patterns and identify patients with increased likelihood of infection caused by MDROs.

Among patients with HA-IAIs, those with post-operative peritonitis may be associated with increased mortality due to underlying patient comorbidity, atypical presentation due to non-specific clinical signs, and risk factors for acquiring MDROs and *Candida* spp. infections [14–16].

Grading of the clinical severity of patients with cIAIs has been well described by the sepsis definitions [17]. The data from WISS study showed that mortality was significantly affected by sepsis status when divided into four categories. Mortality rates increase in patients developing organ dysfunction and septic shock. Mortality by sepsis status was as follows: no sepsis 1.2%, sepsis only 4.4%, severe sepsis 27.8%, and septic shock 67.8% [18].

Recently, the third International Consensus Definitions for Sepsis and Septic Shock (Sepsis-3) were published [19], replacing previous classifications [20, 21]. Sepsis is now defined as a life-threatening organ dysfunction caused by a dysregulated host response to infection. Organ dysfunction can be represented by an increase in the Sequential [sepsis-related] Organ Failure Assessment (SOFA) score of 2 points or more. Septic shock should be defined as a subset of sepsis and should be clinically identified by a vasopressor requirement to maintain a mean arterial pressure of 65 mmHg or greater and serum lactate level greater than 2 mmol/L in the absence of hypovolemia.

Designing a classification which is accepted worldwide may be important to stratify patients according to the risk for antimicrobial therapy failure. A new classification would permit uniform regimens for patients having cIAIs and increase the comparability of studies carried out at different centers.

## What are the optimal methods in diagnosing IAI that balances benefits and risks?

**Statement 2**

**Early clinical evaluation is essential for diagnosing IAIs. It helps to optimize diagnostic testing and can result in earlier implementation of a proper management plan (Recommendation 1C).**

**Statement 3**

**A step-up approach for diagnosis should be used and tailored to the clinical setting, resources, patient’s age beginning with clinical and laboratory examination and progressing to imaging examinations (Recommendation 1C).**

Diagnosis of complicated IAIs is mainly clinical. Early detection and treatment is essential to minimize complications of IAIs [22, 23].

Patients with IAIs typically present with rapid-onset abdominal pain and signs of local and systemic inflammation (pain, tenderness, fever, tachycardia, and/or tachypnea). Hypotension and hypoperfusion signs such as oliguria, acute alteration of mental status, and lactic acidosis are indicative of ongoing organ failure.

Physical evaluation may limit the differential diagnosis to direct decisions regarding a proper management plan including the need for appropriate diagnostic testing, the need for initiation of antimicrobial therapy, and whether emergent intervention is required [22].

The value of physical findings in the diagnostic work-up for IAIs has been studied in relation to acute appendicitis where signs and symptoms are helpful in diagnosing or excluding appendicitis [24].

The presence of a positive psoas sign, fever, or migratory pain to the right lower quadrant suggests an increased likelihood of appendicitis. Conversely, the presence of vomiting before pain makes appendicitis unlikely [25].

Ultrasound (US) and computed tomography (CT) have been used over the last two decades to complete the clinical assessment of patients with IAIs. Although CT has higher sensitivity and specificity [26], concerns about radiation exposure have recently prompted reappraisal of the roles of sonography [27] including performance by surgeons [28].

Proposals of staged algorithms using a step-up approach with CT performed after an inconclusive or negative US have been proposed in the setting of acute appendicitis and acute diverticulitis [29–32].

Magnetic resonance imaging (MRI) is not routinely available in most hospitals in the emergency setting. It has been proposed to be used in pregnant patients with abdominal pain when US is inconclusive [33]. Recently, a systematic review and meta-analysis of diagnostic performance of MRI for evaluation of acute appendicitis was published [34]. A total of 30 studies that comprised 2665 patients were reviewed. The sensitivity and specificity of MRI for the diagnosis of acute appendicitis was 94% (95% CI, 87–98%) and 96% (95% CI, 95–97%), respectively.

Laparoscopy is gaining wider acceptance in emergency surgery [35]. When imaging has been unhelpful, diagnostic laparoscopy may be used to identify the causative pathology of acute abdominal pain followed by definitive laparoscopic treatment. The accuracy of diagnostic laparoscopy is very high reporting definitive diagnosis rates between 86 and 100% in unselected patients [36, 37].

## Which patients are at high risk of failure?

**Statement 4**

**Patient factors are essential when addressing treatment outcome, as advanced age, associated comorbidity, pre-existing disease, and physiologic status greatly influence outcomes (e.g., mortality) (Recommendation 2 C).**

**Statement 5**

**Disease factors are essential to consider when addressing risk for treatment failure. (Recommendation 2 C).**

**Statement 6**

**While age alone is not decisive for outcome it should be recognized that patients with an accumulated number of risk factors, including advanced age, high disease severity and presenting in sepsis or septic shock, have a very high risk of death. Palliative care should be actively discussed when conditions indicate that operative treatment is futile (Recommendation 2 C).**

Defining the patient with IAIs at high risk for failure is difficult. First, it depends on the definition of failure as an outcome; is this mortality? failure of surgical source control? failure of antibiotic control? Various studies use variable definitions of outcome.

Second, “high risk” may be attributed to the patient’s underlying condition(s), such as age, comorbidity or the disease severity status on presentation. In addition, a “low-risk” patient may be converted to “high risk” if the care provider loses the “window of opportunity” to diagnose, resuscitate, and start timely treatment. Thus, there are numerous situations that must be taken into account when addressing high-risk patients and treatment failure.

In general, high-risk IAI is attributed to patient factors (advanced age, immunosuppression, malignant disease, and pre-existing medical comorbidities) or disease factors, represented by high-risk scores (such as ASA, APACHE, SOFA scores), delay in intervention (usually >24 h), inability to obtain source control, and an IAI that is healthcare associated (rather than community acquired) [38].

While age alone is not decisive for outcome, it should be recognized that elderly patients are at high risk for adverse outcomes, including death [39]. Core to the associated poor outcomes is the reduced physiological reserve and limited response to any minor or major stressor that comes with age [40] and which is summarized in the concept of “frailty” [41]. While frailty can be readily defined [41, 42], it is difficult to measure in the emergency setting, although attempted by several recent approximated measures [43–46]. In particular, patients with IAIs and pre-existing malignant disease have a particular high risk for a poor outcome [47, 48].

Disease severity on presentation is strongly associated with risk of failure or poor outcome, including death. The risk is in large part disease-dependent (e.g., lower with appendicitis than for perforated gastroduodenal ulcer); thus, the use of one generic risk score may not be sufficient. For all IAIs, the presence of sepsis or septic shock is a generic prognostic feature and defines a disease process that has become systemic, rather than a localized disease process. Several disease specific scores exist for various organ-related IAIs (appendicitis, diverticulitis, perforated ulcers) [49–52], for which none have good accuracy, prediction ability, nor validity and generalizability.

Pre-admission functional status should be considered [53]; compared to an independent status, nursing home residents have a several-fold increased risk for adverse outcomes, including death.

Patients with an accumulated number of risk factors, including very high age and high disease severity and presenting in sepsis or septic shock, have an excessively high risk of death. The appropriateness of advanced, particularly invasive treatment versus palliative measures should be actively discussed [54, 55].

## Are prognostic scores useful in clinical practice?

**Statement 7**

**Prognostic scoring systems for complicated IAIs may be useful in clinical practise, especially for audit and research. Scoring systems can be broadly divided into two groups: general organ failure severity (ICU) scores and peritonitis-specific (Surgical) scores. The Sequential Organ Failure Assessment (SOFA) score allows physicians to follow the evolving disease process in critically ill patients in ICU (Recommendation 2 A).**

However, although these scores may help guide clinical care, their use in individual patients is much less predictive.

Early prognostic evaluation of cIAIs is important to assess the severity and the prognosis of the disease. It would be valuable to have accurate prognostic scores in IAIs for a variety of reasons which include (1) prediction of prognosis and (2) selection of treatment options/help guide management strategies and allow (3) comparative audit and (4) research, i.e., stratification in clinical trials.

Scoring systems can be broadly divided into two groups:

1. General Organ Failure Severity (ICU) scores
2. Peritonitis-Specific (Surgical) scores

### General Organ Failure Severity (ICU) scores

These scores are used in the ICU and can allow continuous assessment of medical and surgical patients. Over the past years, many scoring models have been developed to describe the severity of illness of intensive care patients or to predict the outcome of intensive care treatment. Some require computer-aided calculations and the input of complex variables, whereas others are more simple. The majority of scores assess various organ systems for the presence of dysfunction (respiratory, hemodynamic, renal, neurological, biochemical, coagulation) and are used in sepsis and other general causes of multiorgan failure.

Severity of illness scoring systems such as the full APACHE II or the Simplified Acute Physiology Score (SAPS) are based on the parameters of the first 24 h of ICU stay and may be applicable also in patients with peritonitis [56]. The SOFA score is a simple and objective severity score that allows both calculation of the number and the severity of organ dysfunction in six organ systems (respiratory, coagulation, liver, cardiovascular, renal, and neurologic) [57]. The SOFA scoring system also tracks the time course of a patient’s condition during the entire ICU stay. Regular, repeated scoring enables patient condition and disease progression to be monitored and better understood. It allows physicians to follow the evolving disease process in critically ill patients [58].

### Peritonitis-Specific (Surgical) scores

These scores are calculated as a one off at the time of surgery and often include intra-operative features of the extent of contamination. Surgical scores can be disease specific; for example, the left-sided colonic Peritonitis Severity Score (PSS) [59]. There are also many scoring systems for perforations of gastroduodenal ulcers, including the Boey Score, Jabalpur Index, Hacettepe Score, and PULP Score [52, 60, 61]. Prognostic scores which can be universally used in a variety of etiologies, include the P-POSSUM, Mannheim Peritonitis Index (MPI), Peritonitis Index Altona (PIA) [62], and WSES complicated IAI score from the WISS study [18]. The MPI score is easy to calculate, even during surgery. It is calculated using simple factors such as degree of peritonitis, age, sex, time from perforation to operation, origin of sepsis, kind of exudates (clear, purulent, or fecal) [63]. Billing et al. demonstrated the reliability of MPI in 2003 patients from seven centers in Europe showing that it is an easy and reliable means of risk evaluation and classification for patients with peritonitis [63].

The WISS study is a multicenter prospective study of 4533 patients published in 2015 [18] specifically to validate the WISS score, which was built on the previous work of the CIAO and CIAOW studies. The WSES/WISS score, estimated at the admission of the patients, includes factors for (1) clinical condition on admission, (2) setting of acquisition, (3) delay in source control, and (4) risk factors including age and immunosuppression. Scores can range from 0 to ≥18. A score above 5.5 was the best predictor of mortality with a sensitivity of 89.2% and specificity of 83.5% (a positive likelihood ratio of 5.4).

However, while all these scores are useful tools for research studies, they are not sufficiently accurate to predict individual patient prognosis. Although it is hoped that these scores may help guide management decisions in the future, at the present time, they are principally used for comparative surgical audit and research tools.

## What is the utility of source control? When should source control be undertaken? Is source control necessary in all patients with cIAI? What approaches should be utilized for source control?

**Statement 8**

**Most patients with cIAIs and sepsis/septic shock should undergo an urgent source control procedure; source control can be delayed in less severely-ill patients when the circumstances are appropriate (Recommendation 2 C).**

Source control encompasses all the measures undertaken to eliminate the source of infection and to control ongoing contamination. Timing and adequacy of source control are currently the most important issues in the management of IAIs because inadequate and late operation may have a negative effect on outcome [64–69]. The optimal timing of source control has not been rigorously investigated. SIS/IDSA guidelines suggest that source control should be performed as soon as possible in patients with diffuse peritonitis but that intervention could be delayed for logistical reasons as long as 24 h in patients with a localized infection if appropriate antimicrobial therapy is given and careful clinical monitoring is provided [22].

In a review by De Waele, source control was considered an essential element in the management of sepsis and should be considered and performed early after the diagnosis is established in most if not all patients [70]. The 2016 Surviving Sepsis Campaign guidelines suggest that a specific anatomic diagnosis of infection requiring emergent source control should be identified or excluded as rapidly as possible in patients with sepsis or septic shock [71].

Sotto et al. in 2002 found in a retrospective study that time between diagnosis and operation was associated with mortality [72]. In this study, the period between diagnosis and surgery (*P* = 0.04) was predictive of death within 30 days after diagnosis of peritonitis emphasizing the importance of prompt surgical treatment. In another retrospective review of 129 patients [73] with non-traumatic severe intra-abdominal infections, multivariate analysis identified that an operating room latency of 60 h or longer (*P* = 0.01) was a predictor of need for relaparotomy.

**Statement 9**

**Highly selected patients with perforated diverticulitis (including those with an abscess <4 cm in diameter), a peri-appendiceal mass, or a perforated peptic ulcer can be managed without definitive source control if responding satisfactorily to antimicrobial therapy and other supportive measures. (Recommendation 1 B).**

An operative intervention remains the most viable therapeutic strategy for managing intra-abdominal sepsis. Surgical source control entails resection or suture of a diseased or perforated viscus (e.g., diverticular perforation, gastroduodenal perforation), removal of the infected organ (e.g., appendix, gallbladder), debridement of necrotic tissue, resection of ischemic bowel, and repair/resection of traumatic perforations with primary anastomosis or exteriorization of the bowel.

However, some data indicate highly selected patients can be managed without definitive source control if responding satisfactorily to antimicrobial therapy alone.

In addition, well-localized fluid collections of appropriate density and consistency (i.e., lack of extensive loculations) may be drained percutaneously with acceptable morbidity and mortality [74–78].

Approximately 15–20% of patients admitted with acute diverticulitis have an abscess on CT scan [79]. These abscesses may be initially treated by intravenous antibiotics alone with or without percutaneous drainage, depending on the size of the abscess. A maximum diameter of 3–6 cm has been generally accepted as a reasonable limit for treatment with antimicrobial therapy alone without drainage [80, 81].

Although the absolute prevalence of perforated diverticulitis complicated by generalized peritonitis is low, it has a significant post-operative mortality, regardless of selected surgical strategy. However, CT findings of distant free air (a known predictor of failure of non-operative treatment) does not necessarily obligate a surgical approach. Dharmarajan et al. [82] described a high success rate for non-operative management in patients with acute diverticulitis and a pneumoperitoneum, excluding those with hemodynamic instability.

Sallinen et al. [83] reported results of non-operative management in patients with CT verified extra-luminal air. This study showed that non-operative treatment was feasible therapy only for hemodynamically stable patients with CT findings of pericolic extra-luminal air or only a small amount of distant intraperitoneal air in the absence of clinical diffuse peritonitis or fluid in the fossa of Douglas. A large amount of distant intraperitoneal air or distant retroperitoneal air even in the absence of clinical generalized peritonitis was associated with a high failure rate (57–60%) of non-operative management.

Recent WSES guidelines for management of acute left colonic diverticulitis [84] state that patients with CT findings of distant air without diffuse fluid may be treated by conservative treatment in selected cases; however, a risk of treatment failure and need for emergency surgery exists.

Antibiotics alone may be used to treat patients with early, non-perforated appendicitis, although there is a significant risk of subsequent recurrence [85].

In patients with complicated appendicitis, presenting with abscess or phlegmon, the optimal management strategy has been somewhat controversial. Several studies have found that non-operative treatment for a peri-appendiceal abscesses or masses results in fewer complications and shorter overall hospitalization [86–88].

In 2010, a meta-analysis [89] was published comparing conservative treatment (i.e., antibiotic therapy with or without percutaneous abscess drainage) to appendectomy for the treatment of complicated appendicitis (cases exhibiting abscess or phlegmon). Seventeen studies (16 non-randomized/retrospective and one non-randomized/prospective) reported clinical data for 1572 patients of whom 847 patients received conservative treatment and 725 underwent acute appendectomy. Conservative treatment was associated with significantly fewer complications, wound infections, abdominal/pelvic abscesses, ileus, bowel obstruction, and additional follow-up surgeries. No significant differences were found in the overall length of hospitalization or in the duration of intravenous antibiotic infusion. Overall, clinical data supports the utility of conservative management rather than appendectomy in selected patients presenting with a peri-appendiceal abscess or phlegmon.

Although the widespread clinical use of H_2_ receptor antagonists, proton-pump inhibitors (PPI), and antibiotic therapy directed against *Helicobacter pylori* has greatly reduced surgical treatment of peptic ulcer disease in recent years, complications, especially perforation, are still frequently seen. Non-operative management of perforated peptic ulcer (PPU) may be an option, although urgent repair of perforation is still the standard approach for PPU in many clinical centers. Several studies have indicated that patients with PPU can be successfully treated non-operatively [90–93]. Non-operative management includes the use of nasogastric drainage, supportive care (IV fluids and nutrition), intravenous antibiotics, intravenous PPI, and radiological drainage of intra-abdominal collections.

The 2013 WSES guidelines for management of IAIs [94] state that in selected cases (age <70 years, no shock, no peritonitis, lack of spillage of the water-soluble contrast medium on gastroduodenogram), non-operative management is an option. However, if no improvement of the patient’s condition is observed within 24 h, surgical therapy is indicated.

## What are the indications for laparoscopic approach to IAIs?

Laparoscopic procedures have become widely accepted by the medical community as a primary means of diagnosing and treating IAIs. On the basis of the anatomical source of infection and the attending surgeon’s experience, laparoscopy may be recommended for the treatment of many IAIs [95].

**Statement 10**

**Laparoscopic appendectomy represents the first choice for most patients with acute appendicitis where appropriate resources and skills are available (Recommendation 1 A).**

Recent WSES guidelines for diagnosis and treatment of acute appendicitis stated that laparoscopic appendectomy (LA) is the first choice where laparoscopic equipment and skills are available [96]. In the Sauerland et al. review, wound infections were less likely after LA than after open appendectomy (OA) (OR 0.43; CI 0.34–0.54); however, the incidence of intra-abdominal abscesses was increased (OR 1.87; CI 1.19–2.93) [97]. Despite evidence showing that LA is safe in pregnancy, advantages are minor (less pain, less infections, less early deliveries) if compared to the risk of fetal loss. Recent data from reviews of comparative studies [98] (599 LA versus. 2816 OA) showed an increased fetal loss for LA, without significant advantages. A population-based analysis on 859 pregnant women [99] with appendicitis confirmed a better outcome for those treated surgically versus non-operative management, while it found no difference in maternal complications between LA and OA. The role of antibiotic treatment instead of surgical removal for acute early uncomplicated appendicitis is currently unknown.

**Statement 11**

**There is no evidence of any significant advantages between laparoscopic and open repair of perforated peptic ulcer (PPU). However, laparoscopy has less postoperative pain and shorter hospital stay (Recommendation 1 A).**

Aside from reduced post-operative analgesic demands, the post-operative outcome of the laparoscopic approach does not differ significantly from that of open surgery. In all reported studies, patients presented with small ulcers and received simple sutures, while many also received an omental patch. There were no studies reporting emergency laparoscopic resection or laparoscopic repair of large ulcers.

A systematic review was published in 2013 [100] including randomized clinical trials comparing laparoscopic surgery versus open surgery for the repair of perforated peptic ulcer using any mechanical method of closure (suture, omental patch, or fibrin sealant).

The authors concluded that laparoscopic surgery results are not clinically different from those of open surgery, but there is clearly a selection bias in the studies performed to date and there is a need of more randomized controlled trials with a larger number of patients.

**Statement 12**

**Early laparoscopic cholecystectomy is safe and feasible in acute cholecystitis and should be the preferred choice in absence of contraindications to pneumoperitoneum, even in high risk patients, where appropriate resources and skills are available (Recommendation 1A).**

Recent 2016 WSES guidelines for the management of acute calculous cholecystitis (AAC) [101] stated that cholecystectomy is the gold standard for treatment of ACC. Laparoscopic approach should initially be attempted except in cases of absolute anesthesiology contraindications or septic shock. Many prospective trials have demonstrated that the laparoscopic cholecystectomy is a safe and effective treatment for ACC [102–106]. A recently published meta-analysis demonstrated that laparoscopic cholecystectomy in ACC [107] is the preferred approach having lower mortality and morbidity, significantly shorter post-operative hospital stay, and reduced rate of pneumonia and wound infections, compared with the open technique. Conversion rate ranged from 8 to 35%.

**Statement 13**

**Laparoscopic lavage is not recommended in Hinchey IV diverticulitis because it can not achieve adequate source control. Laparoscopic lavage is safe and not inferior to sigmoid resection in case of Hinchey III but it is not considered the preferred choice, given the lack of evidence of major benefits (Recommendation 1A).**

**Statement 14**

**Laparoscopic sigmoid resection is feasible and safe in selected patients, hemodynamically stable, without significant comorbidities and with onset of peritonitis <12-24 hours, only if specific advanced laparoscopic colorectal expertise is available (Recommendation 2 C).**

A conservative approach using laparoscopic peritoneal lavage and drainage has been debated in recent years as an alternative to colonic resection. Great debate is still open on this topic, mainly due to the discrepancy and sometime disappointing results of the latest prospective trials such as SCANDIV, Ladies, and DILALA trials [108–110].

A recent meta-analysis showed that laparoscopic lavage is a safe procedure in terms of mortality but is associated with a higher intra-abdominal abscess and re-operation rate during the index admission. No definitive high-level conclusions could be drawn by the available literature at this moment, and further evidence is needed to better define the role of laparoscopic lavage in the management of acute perforated diverticulitis with purulent peritonitis [111].

Recent WSES guidelines for the management of acute diverticulitis in the emergency setting [84] stated that considering the conflicting results laparoscopic lavage as treatment for patients with perforated diverticulitis, Hinchey III disease, may be feasible even if it cannot be considered the treatment of choice.

Laparoscopic sigmoidectomy for diverticulitis has initially been confined to the elective setting. However, laparoscopic sigmoidectomy may be feasible for diverticular peritonitis in the emergency setting also in early cases of type IV diverticulitis. In 2016, a systematic review on laparoscopic sigmoidectomy for diverticulitis in the emergency setting was published [112]. The review included only a small number of patients from four case series and one cohort study (total of 104 patients). High-quality prospective or randomized studies are needed to demonstrate benefits of acute laparoscopic sigmoidectomy compared with open sigmoidectomy for perforated diverticulitis.

## Which is the best relaparotomy strategy?

**Statement 15**

**Planned relaparotomy is not recommended as a general strategy in patients with secondary peritonitis (Recommendation 1A).**

Some patients are prone to persisting IAIs regardless of eradication of the source of infection and timely relaparotomy provides the only surgical option that significantly improves outcome. In these cases, single operation may not be sufficient to achieve source control; thus, relaparotomy may become necessary [113, 114]. In 2007, van Ruler and the Dutch peritonitis group [115] published a randomized, clinical trial addressing two aspects of closed abdominal management, comparing an on-demand versus planned relaparotomy strategy in patients with severe peritonitis who had the fascia formally closed after the initial laparotomy.

In this trial, a total of 232 patients with severe IAIs (116 on-demand and 116 planned) were randomized. In planned relaparotomy group, relaparotomies were performed every 36 to 48 h after the index laparotomy to inspect, drain, lavage, and perform other necessary abdominal interventions for residual peritonitis or new infectious focus. In on-demand relaparotomy group, relaparotomies were only performed in patients with clinical deterioration or lack of clinical improvement with a likely intra-abdominal cause. Patients in the on-demand relaparotomy group did not have a significantly lower rate of adverse outcomes compared with patients in the planned relaparotomy group but did have a substantial reduction in relaparotomies, healthcare utilization, and medical costs. Patients in the on-demand group had shorter median ICU stays (7 versus 11 days; *P* = 0.001) and shorter median hospital stays (27 versus 35 days; *P* = 0.008). Direct medical costs per patient were reduced by 23% using the on-demand strategy.

## When and how do you perform a damage control surgery strategy in patients with severe cIAIs?

**Statement 16**

**There is insufficient evidence to advocate damage control surgery as general strategy in patients with secondary peritonitis (Recommendation 1 C).**

In order to value current evidence on damage control surgery for abdominal surgical emergencies a PubMed/MEDLINE literature review was recently published by Weber et al. [116]. Minimal evidence exists to validate the benefits of damage control surgery in general surgical abdominal emergencies. The evidence over the past decade is limited to 16 studies including a total of 455 patients, of which the majority are retrospective case series. Damage control surgery has been performed for a wide range of indications, but most frequently for uncontrolled bleeding during elective surgery, hemorrhage from complicated gastroduodenal ulcer disease, generalized peritonitis, acute mesenteric ischaemia, and other sources of intra-abdominal sepsis.

The concept of damage control surgery has been accepted by emergency surgeons and may be a logical extension from pathophysiological principles in trauma to hemorrhage and sepsis as life-saving tactic in emergency surgery performed on physiologically deranged patients. However, the benefits of this strategy are not clear from the literature.

**Statement 17**

**Damage control surgery may be an option in selected significantly physiologically deranged patients with ongoing sepsis (Recommendation 2 C).**

Damage control surgery was initially developed in the 1980s by Stone et al. [117] and detailed by Burch et al. in the early 1990s [118]. Damage control surgery strategy was defined as the abbreviated laparotomy for trauma patients with the initial control of surgical bleeding by simple operative techniques such as packing as life-saving techniques. The patient was taken to the ICU where subsequent resuscitation corrected hypothermia, acidosis, and coagulopathy [118]. In severe abdominal sepsis, damage control surgery using an open abdomen strategy may allow early draining of any residual infection and control any persistent source of infection, preventing abdominal compartment syndrome and deferring definitive intervention and anastomosis until the patient is hemodynamically stable and thus better able to heal [119]. The concept of damage control surgery may be easily adapted in patients with advanced sepsis and can incorporate the principles of the Surviving Sepsis Campaign (SSC) [71]. Even if little evidence exists, damage control surgery is employed in a wide range of abdominal emergencies and may be an increasingly recognized life-saving strategy in emergency surgery performed on physiologically deranged patients [119]. However, the role of the OA in the management of severe peritonitis is still being debated [120]. Robledo et al. compared open versus closed abdomen procedures in 40 patients with severe secondary peritonitis. They found no significant difference in mortality rates (55% open versus 30% closed). Nevertheless, the relative risk and odds ratio for death in the closed versus open group (1.83 and 2.85, respectively) led to termination of the study at the first interim analysis. However, in that study, the temporary abdominal closure was accomplished by a sandwich technique with non-absorbable mesh sutured to the fascia. Current clinical guidelines suggest that open abdomen technique should not be used routinely but should be individualized for each patient with abdominal sepsis [121, 122]. However, the benefits of this strategy are not yet clear from the literature.

**Statement 18**

**Temporary abdominal closure using negative pressure therapy (NPT) can be useful to decrease the time to definitive abdominal closure (Recommendation 1 B). Prolonged NPT may increase the risk of enteric fistulae.**

Following re-exploration, the goal is early and definitive closure of the abdomen, in order to reduce the complications associated with an open abdomen, such as entero-atmospheric fistulas, fascial retraction with loss of abdominal wall domain, and development of massive incisional hernias. Early definitive closure is the basis for preventing or reducing the risk of these complications [123, 124] and should be the goal when the patient’s physiological condition allows. The literature suggests a bimodal distribution of primary closure rates. Early closure depends on post-operative intensive care management, and delayed closure depends on the choice of the temporary abdominal closure technique [125]. The first mode is to close within 4–7 days and achieve a high rate of primary closure, the second mode has a delay (20–40 days) having lower overall closure rate [125]. If a surgeon is unable to close the abdomen early, the use of a progressive closure device may be necessary. A systematic review and meta-analysis of the open abdomen and temporary abdominal closure techniques in non-trauma patients was recently published [126]. The search identified 74 studies comprising 4358 patients of whom 3461 (79%) had peritonitis. The best results achieved for delayed fascial closure and reduced the risk of entero-atmospheric fistula were shown for NPT with continuous (mesh-mediated) fascial traction. However, authors stated that the overall quality of the available evidence was poor and that uniform recommendations cannot be made.

To determine whether active negative pressure peritoneal therapy (NPPT) with the ABThera temporary abdominal closure device reduces systemic inflammation after abbreviated laparotomy, a single-center, randomized controlled trial was performed by Kirkpatrick [127]. Forty-five adults with abdominal injury (46.7%) or intra-abdominal sepsis (52.3%) were randomly allocated to the ABThera (*n* = 23) or Barker’s vacuum pack (*n* = 22). The cumulative incidence of primary fascial closure at 90 days was similar between groups. However, the 90-day mortality was less in the ABThera group. Given this potential survival benefit, further well-powered multicenter trials should be conducted to confirm these provocative findings.

## When and how do you perform the peritoneal specimens?

**Statement 19**

**Intraperitoneal specimens for microbiological evaluation from the site of infection are always recommended for patients with HA-IAIs or with CA-IAIs at risk for resistant pathogens (previous antimicrobial therapy) and in critically ill patients (Recommendation 1 B).**

**Statement 20**

**Intraperitoneal specimens should be collected in every re-operation (Recommendation 1 C).**

**Statement 21**

**Appropriate intraperitoneal specimen is peritoneal fluid/tissue collected from the site of infection (Recommendation 1 C).**

**Statement 22**

**Sufficient abdominal fluid/tissue volume (usually at least 1–2 mL of fluid) should be collected and transported to the microbiology laboratory using a transport system that properly handles and preserves the samples to avoid damage or compromise their integrity (Recommendation 1 C).**

**Statement 23**

**At the laboratory the intraperitoneal specimen should undergo Gram stain, aerobic and anaerobic culture, and antibiotic susceptibility testing (Recommendation 1 C).**

Initial antibiotic therapy for IAIs is empirical in nature because microbiological data (culture and susceptibility results) may require >24 h before they are available for a more detailed analysis. IAIs are often polymicrobial in etiology. In polymicrobial infections, first the particular bacterial strains have to be isolated and identified from intraperitoneal specimen and after that the antibiotic susceptibility test can be performed. Isolation and identification of bacterial strains take more time, and results of antibiotic susceptibility are usually only available after 48 h and later.

However, the results direct expansion of antimicrobial regimen if it is too narrow and to perform a de-escalation if it is too broad [128, 129], particularly in critically ill patients where de-escalation strategy is one of the cornerstones of antimicrobial stewardship programs [129]. A lack of impact on patient outcomes by bacteriological cultures has been documented in patients with community-acquired IAIs, especially in appendicitis [130, 131]. Microbiological identification in CA-IAIs rarely influences the individual patient management. However, routine bacteriological culture and antibiotic susceptibility test in patients with CA-IAIs help to detect epidemiological changes in the resistance patterns of microbial pathogens associated with CA-IAIs. The results of microbiological testing may have great importance for the choice of therapeutic strategy of every patient at risk for antimicrobial resistance [132]. The recommended initial antibiotic regimen for risk patients should be determined according to regional epidemiological data and resistance profile.

The major pathogens involved in CA-IAIs are likely to be due to a patient’s own flora and are generally predictable and include *Enterobacteriaceae* (predominantly *Escherichia coli* and *Klebsiella* spp.), viridans group streptococci, and anaerobes (especially *Bacteroides fragilis*). In addition, *Enterococcus* spp. are frequently isolated Gram-positive organisms in CA-IAIs with reported percentages of 7.7–16.5% [133].

In 2012, the Dutch Peritonitis Study Group analyzing all patients from the RELAP trial found that the presence of Gram-positive cocci, predominantly *Enterococcus* spp., was associated with worse outcome, although in secondary peritonitis, microbial profiles did not predict ongoing abdominal infection after initial emergency laparotomy [134].

The main current resistance problem is represented by Extended-spectrum beta-lactamases (ESBLs) producing *Enterobacteriaceae*, which are frequently found in CA-IAIs [135]. Specific risk factors for ESBLs phenotype among infecting pathogens includes recent exposure to antibiotics (particularly beta-lactams or fluoroquinolones) within 90 days of IAIs or known colonization with ESBL-producing *Enterobacteriaceae.* In all HA-IAIs, causative pathogens are less predictable; thus, peri-operative cultures are routinely indicated [136, 137].

In a recent retrospective study, data from 242 patients with secondary peritonitis (88 community acquired, 154 post-operative cases) treated in ICU were obtained. Enterococci were isolated in 47.1% of all patients, followed by *E. coli* (42.6%), and other *Enterobacteriaceae* (33.1%), anaerobes (29.8%), and *Candida* spp. (28.9%). The susceptibility rates and spectrum adequacy rate were lower in post-operative than in community-acquired cases. The spectrum adequacy rate of the most common regimen used for secondary peritonitis in the study center, cefotaxime plus metronidazole, was significantly different for community-acquired and post-operative peritonitis episodes, mainly for cases with polymicrobial positive cultures [138].

Recently, Montravers et al. who evaluated the dynamic change of microbial flora in persistent peritonitis [12] observed a progressive shift of peritoneal flora with the number of reoperations. The proportion of patients harboring MDRO strains increased from 41% at index surgery to 49% at the first, 54% at the second (*P* = 0.037), and 76% at the third re-operation (*P* = 0.003 versus index surgery). Intraperitoneal specimens should be collected in every re-operation and re-intervention.

Appropriate intraperitoneal specimen is peritoneal fluid or pus that is collected from the site of infection. For specimen collection, intraperitoneal fluid, pus, or tissue should be collected in airless sterile syringe with combi-stopper or in sterile test tube. Intraperitoneal specimen can be directly inoculated into appropriate aerobic and anaerobic transport media. Peritoneal swab and fluid from drain tubes are not recommended routinely. Sufficient fluid volume (usually at least 1–2 mL of fluid or tissue) must be collected, and then transported to the microbiology laboratory using a transport system that properly handles and preserves the samples to avoid damage or compromising their integrity [132].

At the laboratory, the intraperitoneal specimen should be examined under a microscope using Gram stain, aerobic and anaerobic culture should be established, and antibiotic susceptibility test should be performed. Received peritoneal fluid or pus may be examined under the microscope immediately because Gram stains may be of value in detecting Gram-positive cocci or yeast. In the event of positive finding of Gram-positive cocci or yeast, the additional empiric anti-cocci or anti-fungi therapy should be considered before definitive culture results are available.

When a microorganism is identified in clinical cultures, antimicrobial susceptibility testing (AST) should always be performed to guide antibiotic therapy.

Results are reported as the minimal inhibitory concentration (MIC), which is the lowest concentration of drug that inhibits the growth of the organism. Reports typically contain a quantitative result in μg/mL and a qualitative interpretation. The interpretation usually categorizes each result as susceptible (S), intermediate (I), and resistant (R) basing on breakpoints established by either the Clinical or Laboratory Standards Institute (CLSI) in the USA or the European Committee for Antimicrobial Susceptibility Testing (EUCAST) in Europe [132].

## What are the principles of antimicrobial therapy?

**Statement 24**

**Empirical antimicrobial therapy should be based on local epidemiology, individual patient risk factors for difficult to treat pathogens, clinical severity of infection, and infection source (Recommendation 1 C).**

The principles of empiric antibiotic treatment should be defined according to the most frequently isolated microbes, always taking into consideration the local trend of antibiotic resistance.

In this era of prevalent drug-resistant microorganisms, the threat of resistance is a source of major concern that cannot be ignored [139].

In the past 20 years, the incidence of IAIs caused by MDROs has risen dramatically [132].

Quinolone resistance, prevalence of ESBL-producing bacteria, prevalence and mechanisms of carbapenem resistance in the local environment and the place of recent traveling should be always taken into account when an antimicrobial therapy is administered empirically.

Generally, the most important factors in predicting the presence of resistant pathogens in IAIs is acquisition in a healthcare setting (particularly if the patient becomes infected in the ICU or has been hospitalized for more than 1 week), corticosteroid use, organ transplantation, baseline pulmonary or hepatic disease, and previous antimicrobial therapy [136].

Previous antimicrobial therapy is one of the most important risk factors for resistant pathogens [15].

In order to determine risk factors for presence of MDROs in post-operative peritonitis (PP), Augustin et al. reviewed data of 100 patients hospitalized in the ICU for PP. According to logistic regression analysis, the use of broad-spectrum antibiotic between initial intervention and re-operation was the only significant risk factor for emergence of MDROs [15]. For patients with severe sepsis or septic shock, early and properly administered empirical antimicrobial therapy can have a significant impact on the outcome, independent of the anatomical site of infection. Riché et al. [140] in a prospective observational study involving 180 consecutive patients with secondary generalized peritonitis demonstrated a significantly higher mortality rate for patients in septic shock (35 and 8% for patients with and without shock, respectively). Clinical severity should be assessed with the sepsis score criteria or the new proposed criteria for definition of sepsis [19].

Inappropriate therapy in critically ill patients may have a strong negative impact on outcome. An ineffective or inadequate antimicrobial regimen is one of the variables more strongly associated with unfavorable outcomes in critically ill patients. Broad empiric antimicrobial therapy should be started as soon as possible in patients with organ dysfunction (sepsis) and septic shock [141–145].

International guidelines for the management of sepsis and septic shock (the Surviving Sepsis Campaign) recommend intravenously administered antibiotics as soon as possible and in any case within the first hour of onset of sepsis and the use of broad-spectrum agents with adequate penetration of the presumed site of infection [71].

Finally, selection of empirical therapy should take into account the infection source because etiological distribution varies according to the source site, with a prevalence of Gram positive and Candida in the high gastrointestinal tract and a progressive increase of anaerobes and Gram negative towards the lower gastrointestinal tracts.

The epidemiology of intra-abdominal flora in critically ill patients with secondary and tertiary abdominal sepsis was studied by de Ruiter et al. [146]. A 5-year prospective observational cohort study was performed in patients admitted to the ICU with abdominal sepsis syndrome, defined as a perforation of the digestive tract and inflammatory response with organ failure. Abdominal fluid was taken for microbial culture from 221 of the 239 patients admitted with abdominal sepsis. Aerobic Gram-negative bacteria (AGNB) were found in 53% of the cultures of the abdominal fluid taken at the time of operation, of which 45% were *E. coli*; in 36% of patients, more than one AGNB was found. The incidence of AGNB was the highest in colorectal perforations (68.6%) and perforated appendicitis (77.8%) and the lowest in gastroduodenal perforations (20.5%). Gram-positive bacteria were found in 42.5% of the abdominal fluid cultures and most frequently in colorectal perforations (50.0%). Candida was found in 19.9% of patients, with 59.1% of these cultures being *Candida albicans.* The incidence of *Candida* was 41.0% in gastroduodenal perforations and 11.8% in colorectal perforation. Anaerobic bacteria were cultured in 77.8% of patients with perforated appendicitis. Over time, the prevalence of AGNB in abdominal fluid decreased from 117 patients (52.9%) in the first culture to one patient (6.7%) in week 4 (efficacy 87%). The prevalence of Gram-positive bacteria increased from 42.5 to 86.7% in a 4-week period.

**Statement 25**

**The correct dose and correct administration of antimicrobials should include: 1) loading dose when indicated, especially in critically ill patients; 2) extended or prolonged infusion for beta-lactam antibiotics; 3) peritoneal distribution (Recommendation 2 C).**

To ensure timely and effective administration of antimicrobial therapy in patients with severe infection, clinicians must consider also the pharmacokinetic/pharmacodynamics properties of the employed antibiotics.

The “dilution effect,” also known as the “third spacing phenomenon,” is very important for hydrophilic agents. Higher than standard loading doses (LD) of hydrophilic agents such as beta-lactams should be administered to ensure optimal exposure at the infection site, maintaining a therapeutic threshold that considers the effects of renal function [94].

In patients with sepsis and septic shock, missing of LD results in an under exposure to hydrophilic antibiotics that may be critical for patients [147, 148].

Once appropriate initial loading is administered, daily reassessment of the antimicrobial regimen is warranted because pathophysiological changes may significantly affect drug availability [132].

The mechanisms of action of antibiotics should also be taken into account in establishing the maintenance dose. Some antibiotics, including beta-lactam antibiotics, exhibit time-dependent activity and exert optimal bactericidal activity when drug concentrations are maintained above the Minimum Inhibitory Concentration (MIC), whereas high peak concentrations are not beneficial. As a consequence, the more prolonged the time during which the drug levels are above the MIC value, the greater is the chance of clinical cure. In fact, prolonged or continuous infusions of beta-lactams have been proposed in order to maximize the time that the drug concentration exceeds the MIC.

However, the results obtained from clinical studies on the continuous or extended infusion of beta-lactams, including two blinded multicenter RCTs, were not conclusive [149, 150]. The first RCT, of 60 patients, showed that continuous infusion (CI) was associated with higher clinical cure, while the second one, of 432 patients, did not find significant differences in the clinical cure of patients receiving CI or intermittent administration of beta-lactams. This difference could be partially explained by the enrollment of patients on renal replacement therapy in the second RCT.

In addition, these results may not be generalizable to patients with high severity of illness and infections caused by less susceptible pathogens with high MIC for which the greatest potential for a clinically relevant benefit may be predicted by pharmacokinetic/pharmacodynamic theory. This has been supported by some retrospective studies [151, 152]. Prolonged or continuous infusions of beta-lactams should therefore be considered for the treatment of critically ill patients with HAI-IAIs.

Conversely, for antibiotics with concentration dependent activity, such as aminoglycosides, the use of a higher dose at extended interval (i.e., once daily) is strongly recommended [153].

The use of therapeutic drug monitoring (TDM) has been associated with higher clinical success and lower rate of toxicity. It is recommended mainly, but not only, for drugs with a narrow ratio between efficacy and toxicity, such as glycopeptides and aminoglycosides [154].

Antimicrobials typically need to reach a site of action outside the plasma passing through the capillary membranes. Disease- and drug-related factors contribute to differential tissue distribution [155]. Concentration gradient between the plasma and the peritoneal space may be of high relevance in case of multidrug-resistant bacteria. It has been studied for some antibiotics and has shown large variability in drug availability. Data suggest that increased doses of ceftazidime, meropenem, and imipenem are required to reach adequate concentrations in patients with severe intra-abdominal infections [156–158]. In contrast, plasma and peritoneal concentrations of cefepime and cefotaxime seem to be quite similar and an increase in the doses is probably not required [159, 160]. The same concept can be applied for tigecycline with lipophilic characteristics that are minimally impacted by severe intra-abdominal infections [161].

**Statement 26**

**The patient should be reassessed when the results of microbiological testing are available. Antimicrobial de-escalation or withdrawal should be considered (Recommendation 1C).**

The results of microbiological testing may have great importance for the choice of therapeutic strategy of every patient, in particular in the adaptation of targeted antimicrobial treatment.

They provide an opportunity to expand antimicrobial regimen if the initial choice was too narrow but also allow de-escalation of antimicrobial therapy if the empirical regimen was too broad.

Antibiotic de-escalation has been associated with lower mortality rates in ICU patients and is now considered a key practice for antimicrobial stewardship purposes [129].

Recently, Montravers et al. [128] valued the characteristics and outcomes of anti-infective de-escalation during healthcare-associated IAIs. They demonstrated that de-escalation is a feasible option in patients with polymicrobial infections such as HAI-IAIs. However, MDR non-fermenting Gram-negative organisms limit its implementation in the setting of IAIs.

**Statement 27**

**In the settings with a high incidence of ESBL-producing Enterobacteriaceae, the extended use of cephalosporins should be discouraged and should be limited to pathogen-directed therapy because of its selective pressure resulting in emergence of resistance (Recommendation 1C).**

In the past, cephalosporins have often been used in the treatment of IAIs. In light of the increasing prevalence of ESBL-producing *Enterobacteriaceae* and MRSA due to selection pressures related to overuse of cephalosporins, the routine use of these antibiotics should be strongly discouraged [162].

Ceftolozane/tazobactam [162, 163] and ceftazidime/avibactam [164, 165] are two new cephalosporins/beta-lactamase inhibitor combinations approved for IAIs.

By adding beta-lactamase inhibitor (tazobactam or avibactam), ceftozolane and ceftazidime have a strong activity against Gram-negative MDROs including ESBL-producing Enterobacteriaceae.

These new agents should be combined with metronidazole for complicated IAIs due to limited activity against some *Bacteroides spp*. These antibiotics will be valuable for treating infections caused by Gram-negative MDROs in order to preserve carbapenems. Notably, ceftazidime/avibactam has demonstrated consistent activity against *Klebsiella pneumoniae* carbapenemases (KPCs) producers and ceftolozane/tazobactam against multiresistant *Pseudomonas*.

Cautious clinical use is advised, until their precise roles are further defined as empirical treatment.

**Statement 28**

**Extended use of fluoroquinolones (FQ) should be discouraged because of its selective pressure (mainly ESBLs producing Entrobacteriaceae and MRSA). They should be generally used in patients with allergy to beta-lactams (Recommendation 1C).**

FQ have been the most commonly used regimen for complicated IAIs in recent years.

Except for moxifloxacin, FQ have a moderate activity against anaerobes and have been used in combination with metronidazole in the empiric treatment of IAIs. In recent years, resistance of *E. coli* to FQ has risen over time [166].

The worldwide increase in FQ resistance among *E. coli* and other *Enterobacteriaceae* has limited the non-stratified use of FQ for empirical treatment of IAIs, particularly in critically ill patients and those with HAI-IAIs.

Ciprofloxacin, in combination with metronidazole, should be administered only in patients with mild infection and without risk factors for difficult to treat pathogens (DTTPs). In these patients, moxifloxacin monotherapy could be an option [167, 168].

## What is the impact of the antimicrobial resistance in IAIs. How do you treat it?

**Statement 29**

For patients with CA-IAIs, agents with a narrower spectrum of activity should be suggested. However, according to local ecology anti-ESBL-producer coverage may be warranted. By contrast, for patients with HA-IAIs, antimicrobial regimens with broader spectra of activity are preferred (Recommendation 1B).

Hospital-acquired IAIs are associated with an increased likelihood of pathogens with reduced susceptibility to standard antibiotic regimens. In particular, the group of patients with IAIs suffering from resistant bacteria frequently comprises the entire group of post-operative and tertiary peritonitis. According to local bacterial ecology, resistant pathogens can be frequently isolated even in community-acquired secondary peritonitis [170].

In the context of IAIs, the main resistance problem is posed by ESBL-producing *Enterobacteriaceae*, which are alarmingly prevalent in nosocomial infections and frequently observed in community-acquired infections [169–171]. A Carbapenem-sparing regimen is preferred.

The Study for Monitoring Antimicrobial Resistance Trends (SMART) program monitors the activity of antibiotics against aerobic Gram-negative IAIs.

The prevalence of ESBLs in patients with IAIs has increased over time in Asia, Europe, Latin America, the Middle East, North America, and the South Pacific. In contrast, the trend for ESBLs in intra-abdominal infection isolates from Africa has surprisingly, statistically decreased over time [172].

KPC producers are rapidly emerging as a major source of multidrug-resistant infections worldwide. The recent emergence of carbapenem resistance among *Enterobacteriaceae* poses a considerable threat to hospitalized patients [173]. Inappropriate use of carbapenems should be avoided to reduce their selective pressure and association with the increase in carbapenem-resistant *Enterobacteriaceae* (CRE) [174].

**Statement 30**

**Carbapenem sparing treatment should be recommended particularly in the settings where there is a high incidence of carbapenem resistant K. pneumoniae (Recommendation 1B).**

The available therapeutic options for the multiresistant Gram-negative bacteria are limited.

In recent years, clinicians have become dependent on carbapenems for treating ESBL infections. This emphasizes the importance of carbapenem-preserving antimicrobial stewardship [162].

The role of β-lactam/β-lactamase inhibitor (BLBLI) combinations towards ESBLs has been debated and controversial even if recent reports suggest their use in ESBL infections [175, 176].

Although tigecycline does not feature in vitro activity against *P. aeruginosa* or certain *Enterobacteriaceae* (*Proteus* spp., *Serratia* spp., *Morganella morganii*, *Providencia stuartii*), it is still an option for complicated IAIs because of its favorable in vitro activity against anaerobic organisms, enterococci, several ESBLs, and some strains of carbapenemase-producing *Enterobacteriaceae* [177, 178]. Because of poor plasma concentrations, tigecycline performs poorly in bacteremic patients, with a much higher risk of failing to clear bacteremia [179]. Tigecycline should not be considered as first-line therapy in patients with healthcare-associated pneumonia and bacteremia.

The recent challenges in the management of Gram-negative MDROs infections, especially in critically ill patients, have revived the clinical use of polymyxins [180–182] and fosfomycin [183]. There are still open questions about the need of combination therapy and the role of carbapenems, administered at high doses and by extended infusions, in the treatment of infections with carbapenem-resistant entetobacteriaceae.

Ceftolozane/tazobactam and ceftazidime/avibactam have recently been approved in some national agencies for the treatment of IAIs [162–165]. Ceftolozone/tazobactam is a new antibiotic that has been approved for treatment of cIAIs (in combination with metronidazole) including infection by ESBLs and *P. aeruginosa* and associated with metronidazole may be valuable for treating infections caused by Gram-negative MDROs in order to preserve carbapenems [162, 163]. It may be useful as empirical therapy to preserve the use of carbapenems in critical patients with risk factors for ESBL isolation or as targeted therapy in patients with isolation of an ESBL-producing *enterobacteriaceae* or *P. aeruginosa* MDR. It should be considered that in some countries, the production of metallo*-β-*lactamase*s* enzymes, that are not inactivated by ceftolozane/tazobactam, may be one of the mechanisms for carbapenem resistance in *P. aeruginosa.*

**Statement 31**

**Antimicrobial resistance among enterococcal isolates (ampicillin, gentamcin or vancomycin resistance) is mostly found in nosocomial (postoperative or tertiary) peritonitis. In Vancomycin-resistant Enterococcus (VRE), treatment with linezolid (monomicrobial infection) or tigecycline (polymicrobial infection) is appropriate (Recommendation 1 B).**

Among Gram-positive bacteria, Enterococci play a significant role in IAIs. Although they are found in community-acquired infections, they were far more prevalent in hospital-acquired infections [184]. In the CIAOW Study [169], Enterococci (*E. faecalis and E. faecium*) were the most prevalent bacteria among all the aerobic Gram-positive bacteria isolated in the intra-operative samples, representing 15.9% of all aerobic isolates. Although Enterococci were also present in community-acquired infections, they were more prevalent in HA-IAIs (22.3% in HA-IAIs versus 13.9% in CA-IAIs). Some studies have demonstrated poor outcomes among patients with documented enterococcal infections [185–188], particularly in those with post-operative IAIs where Enterococci coverage should always be considered. Empirical coverage of Enterococci is not generally recommended for patients with CA-IAIs. The acquisition of glycopeptide resistance by enterococci has seriously affected the treatment and control of these organisms. Many factors can increase the risk of vancomycin-resistant Enterococcus (VRE) infection. These include previous antibiotic therapy, prolonged hospitalization, hospitalization in an intensive care unit, severe illness or underlying pathology, invasive procedures, gastrointestinal surgery, organ transplantation, and close proximity to other VRE-positive patients [189].

The majority of vancomicyn-resistant enterococcus infections have been attributed to *E. faecium*, though glycopeptide resistance occurs in *E. faecalis* and other *Enterococcus* spp. as well [189].

Options for treating vancomicyn-resistant enterococcus infections are linezolid or tigeciclyine [178, 190].

## What is the impact of intra-abdominal candidiasis? When and how do you treat intra-abdominal candidiasis?

**Statement 32**

**The presence of Candida spp. in the peritoneal samples is a factor of poor prognosis (Recommendation 1C).**

The epidemiological role of *Candida* spp. in IAI has not yet been conclusively defined.

Colonizing strains of *C. albicans* are normally present in small or moderate numbers and they are only regarded as part of the resident gastrointestinal microflora [191].

However, isolation of *Candida* spp. in samples from IAIs is associated with poor outcomes [192].

In community-acquired infections, the role of *Candida* spp. in the prognosis is difficult to demonstrate. In healthcare-associated (mainly post-operative) peritonitis, intra-abdominal candidiasis is associated with increased mortality.

In an observational study, Montravers et al. [193] showed that isolation of *Candida* spp. was an independent risk factor of mortality in nosocomial peritonitis patients. However, antifungal treatment did not improve survival.

Several criteria have been considered as factors of poor prognosis for patients with Candida isolation in peritoneal fluid.

In a retrospective review of a prospective surgical ICU database of patients, Dupont et al. [194] demonstrated that four variables were independently associated with mortality in polymicrobial peritonitis with Candida isolation in peritoneal fluid in critically ill patients. These were (1) APACHE II (Acute Physiology and Chronic Health Evaluation II) score on admission of at least 17, (2) respiratory failure on admission, (3) upper gastrointestinal tract origin of peritonitis, and (4) results of direct examination of peritoneal fluid that were positive for Candida.

Data from 13 hospitals in Italy, Spain, Brazil, and Greece over a 3-year period (2011–2013) including patients from ICU, medical, and surgical wards with intra-abdominal candidiasis was published in 2015 [195]. A total of 481 patients were included in the study. Of these, 27% were hospitalized in ICU. The mean age was 63 years, and 57% of patients were male. Multivariate logistic analysis regression showed that age, increments in 1-point APACHE II scores, secondary peritonitis, septic shock, and non-adequate abdominal source control were significantly associated with mortality. In patients with septic shock, absence of source control resulted in mortality rates above 60% irrespective of administration of an adequate antifungal therapy.

Moreover, a prospective observational study involving 180 consecutive patients with secondary generalized peritonitis (community-acquired and post-operative) at a single center was published by Riché et al. [140]. Septic shock complicating intra-abdominal candidiasis was associated with high mortality rates. Yeast in the peritoneal fluid of post-operative peritonitis were an independent risk factor for death in patients with septic shock.

**Statement 33**

**Two situations justify an empirical antifungal therapy: patients with septic shock in community-acquired infections or patients with post-operative infections (Recommendation 2 C).**

No study has specifically evaluated the efficacy of antifungal therapy in IAIs. In recent randomized trials focusing on antifungal therapy of invasive candidiasis, the proportion of patients with a diagnosis of abdominal candidiasis was low [196, 197]. The need for an early adequate systemic antifungal therapy in candida peritonitis is based on the assumption that delayed antifungal therapy initiation is associated to poorer outcome, particularly among those with candidaemia [198–200]. However, a deleterious impact of delayed systemic antifungal therapy initiation has never been demonstrated in candida intra-abdominal infection.

Two situations justify an empirical antifungal therapy: patients with septic shock in community-acquired infections or patients with post-operative infections where the presence of yeast is associated with a poor prognosis.

The EUCAST guidelines consider *Candida glabrata* resistant to azole agents [201]. These organisms were observed in 22% of all intra-abdominal candidiasis in Montravers et al.’s prospective study. This was a non-interventional study in 271 adult ICU patients with proven invasive *Candida* infection who received systemic antifungal therapy [193]. As a consequence, an echinocandin should probably be used as empirical antifungal therapy in critically ill patients having CA-IAIs or HAI-IAIs. First-line fluconazole therapy is preferable in the other cases. The optimal duration of definitive treatment is not established. The IDSA guidelines did not provide any recommendations for duration of therapy [202]. For severe bacterial healthcare-associated IAIs, a duration of antibacterial therapy between 7 and 15 days is recommended [203]. Based on the high rates of recurrence and relapse in candida IAIs, longer duration has been recommended by experts (around 2 to 3 weeks) [195]. De-escalation of empirical antifungal therapy is a safe procedure as illustrated recently in two studies. In a recent study, 835 non-neutropenic adults were recruited in the multicenter prospective observational AmarCAND2 study. Patients receiving systemic antifungal therapy for a documented or suspected invasive candidiasis in the ICU and who were still alive 5 days after antifungal initiation were selected [204]. Among the 647 studied patients, early de-escalation at day 5 after antifungal initiation occurred in 142 patients (22%). Early systemic antifungal therapy (SAT) de-escalation was the sole factor not associated with increased 28-day mortality (RR 1.12, 95% CI 0.76–1.66). In non-neutropenic critically ill adult patients with documented or suspected invasive candidiasis, SAT de-escalation within 5 days was not related to increased day 28 mortality but was associated with decreased SAT consumption. These results suggest, for the first time, that SAT de-escalation may be safe in these patients. In a recent study by Montravers et al. [126] on 206 patients with HCAI-IAIs, de-escalation was performed in 53% of the cases including antifungal agents in 49% of the cases having antifungal therapy. De-escalation was not a risk factor for mortality.

## What is the optimal duration of antimicrobial therapy?

**Statement 34**

**In the setting of uncomplicated acute cholecystitis and acute appendicitis post-operative antimicrobial therapy is not necessary (Recommendation 1A).**

In the event of uncomplicated IAIs, the infection involves a single organ and does not extend to the peritoneum. When the source of infection is treated effectively by surgical excision, post-operative antimicrobial therapy is not necessary, as demonstrated in managing uncomplicated acute appendicitis or cholecystitis [3–5].

**Statement 35**

**In patients with IAIs, when patients are not severely ill and when source control is complete, a short course (3–5 days) of post-operative therapy is suggested (Recommendation 1A).**

Recently, a prospective study on appropriate duration of antimicrobial therapy was published [6]. The study randomized 518 patients with IAIs and adequate source control to receive antibiotics until 2 days after the resolution of fever, leukocytosis, and ileus, with a maximum of 10 days of therapy (control group), or to receive a fixed course of antibiotics (experimental group) for 4 ± 1 calendar days.

In patients with intra-abdominal infections who had undergone an adequate source control procedure, the outcomes after fixed-duration antibiotic therapy (approximately 4 days) were similar to those after a longer course of antibiotics (approximately 8 days) that extended until after the resolution of physiological abnormalities. In this study, most patients were not severely ill.

**Statement 36**

**In patients with ongoing or persistent IAIs, the decision to continue, revise, or stop antimicrobial therapy should be made on the basis of clinician judgment and laboratory information (Recommendation 1 A).**

The high mortality associated with abdominal sepsis requires clinicians to maintain a high index of clinical suspicion of treatment failure and the early diagnosis of ongoing infections. These patients should always be monitored carefully including the potential use of inflammatory response markers.

The most studied biomarkers in clinical settings are the acute phase proteins (CRP) and procalcitonin (PCT) [205, 206].

Recently, PCT has been suggested as a novel biomarker that may be useful in guiding therapeutic decision making in the management of sepsis. It may be a helpful tool to determine the timing and appropriateness of escalation of antimicrobial therapy in sepsis. A systematic review and meta-analysis on PCT-guided therapy in ICU patients with severe sepsis and septic shock was published in 2013 [207]. Seven randomized studies comprising a total of 1075 patients with severe sepsis or septic shock were included in the meta-analysis. Both hospital mortality and 28-day mortality were not different between PCT-guided therapy and standard treatment groups. However, duration of antimicrobial therapy was significantly reduced in those using PCT-guided therapy. In 2015, a review on PCT-guided antibiotic therapy for septic patients in the surgical ICU confirmed the benefits of using procalcitonin including cost-effectiveness and timing of termination of antibiotics [208].

However, these results were not confirmed in a prospective study in the setting of patients having only IAIs [209]. In this study including 101 consecutive patients with peri-operative septic shock secondary to intra-abdominal infection, PCT decrease to 0.5 ng/mL lacked sensitivity to predict the treatment response. In addition, a decrease of at least 80% from the peak level failed to accurately predict treatment response. PCT may become a valuable weapon for predicting treatment response. However, its role has not been defined in cIAIs and should always be correlated with clinician judgment.

## Which interventions improve antibiotic prescribing practices for patients with IAIs?

Interventions to improve antibiotic prescribing practices for patients with IAIs should be directed at two different levels:

1. Patient level—which includes clinical severity, epidemiological exposures, PK/PD factors, comorbidities, prior antibiotic exposure, prior infection, or colonization with MDROs and infection source
2. Hospital level—including presence of in-hospital antimicrobial stewardship programs, availability of local guidelines and updated microbiological data, infection control policy, educational activities, and structural resources (like computer-assisted order entry)

Significant data supports the importance of antibiotic prescribing practices for patients with IAIs, in critically ill and non-critically ill patients and in community and hospital-acquired infections. Prescribing practices may influence the outcome and cost of treatment as well as the risk of superinfection and resistant pathogens in the individual patient and the broader environment. Components of antibiotic prescribing practices that may influence outcome and the risk of developing superinfection and antibiotic resistant infections include (a) adequacy of empiric antibiotic therapy, (b) the time to initial antibiotic therapy, (c) appropriate pharmacokinetic dosing, (d) de-escalation of antibiotic therapy, (e) length of treatment, and (f) avoidance of unnecessary antibiotic therapy.

**Statement 37**

**Multifaceted interventions are more likely to improve antibiotic prescribing practices than simple, passive interventions. Didactic educational programs alone are generally ineffective. (Recommendation 1B).**

Most studies of the implementation of guidelines and prescribing practices, including for IAIs, have involved multifaceted interventions [210–214]. A longitudinal study of a multifaceted program that included locally developed, unit-specific protocols, computer-assisted order entry, and ICU-based pharmacist facilitation in both a trauma and a surgical ICU, demonstrated compliance with protocols and a sustained reduction in multidrug-resistant pathogens [212]. Popovski et al. performed a before-and-after study that examined multifaceted interventions to optimize antibiotic use for IAIs [214]. Interventions included (1) adapting published guidelines based on local susceptibility data with stratification of infection type, (2) creation of educational tools, and (3) educational programs involving multidisciplinary groups. Antibiotic selection was significantly altered, and the change persisted for greater than 2 years. However, no change was noted in the length of treatment. The difficulty of altering prolonged antibiotic therapy is highlighted by a recent randomized, controlled trial of a short, fixed duration of antibiotic therapy for IAIs versus treatment until the resolution of fever, leukocytosis, and ileus in which both the control and treatment groups had substantial non-compliance with significant extended antibiotic therapy (24 and 18%, respectively) [6].

**Statement 38**

**As a single intervention, implementation of locally adapted, interdisciplinary evidence based guidelines that incorporate risk stratification (severity and CA-IAIs versus HA-IAIs) and local resistance data most consistently improves components of AB prescribing for IAI (Recommendation 1 B).**

As an individual component of change, the creation of locally adapted evidence-based guidelines incorporating local historical culture data with the involvement of an interdisciplinary team is strongly supported in the literature [210, 213, 214]. The presence of pre-existing locally developed antibiotic protocols was independently associated with improved time to antibiotic treatment and with survival in a 1-month prospective observational trial of 41 French ICUs [215]. An intra-abdominal source of infection accounted for 21% of all infection episodes receiving new antibiotic therapy. Several studies have demonstrated the ability to achieve a high percentage of adequate empiric coverage for IAIs employing this approach (Leone 89%, Raymond 100%, Barie 94%) [216–218].

**Statement 39**

**Computer-assisted order entry, non-physician healthcare provider facilitation, and therapeutic drug monitoring can improve components of antibiotic prescribing practices, if resources are available (Recommendation 2 B).**

Computerized decision support programs and computer-assisted implementation methods have been found to be useful in many studies of antibiotic therapy [219–221]. In one cluster randomized trial of three hospitals from Israel, Germany, and Italy, a computerized decision support system significantly increased the rate of appropriate empirical antibiotic treatment of infections, with an adjusted odd ratio of 1.48 [222]. Computer-based advisors and decision support have also been used to improve antibiotic dosing in various populations.

Though seldom examined in isolation, non-physician healthcare providers, most commonly pharmacists and nurses, have facilitated guideline implementation and compliance in most randomized trials and before-and-after studies [223–225].

## Which are the determinants of outcome of patients with cIAIs in ICU?

**Statement 40**

**The lack of source control and antibiotic adequacy are the only modifiable risk factors for mortality in patients with cIAIs admitted to the ICU. Organ dysfunction is associated with worse outcomes (Recommendation 1 B).**

Patients with cIAIs present with varying severity of illness and may require ICU care in the pre or post-operative periods which may be associated with a high mortality rate.

Although cIAIs are the second most frequent infection in the ICU, outcomes after ICU admission and risk factors have been sparsely studied.

De Waele et al. performed a systematic review of the literature to identify factors independently associated with outcome in patients admitted to the ICU because of cIAIs. Studies were included in the analysis if they include ICU patients with cIAIs and reported the impact of any clinical treatment or microbiological factor on outcome. Thirty-two studies, published between 1999 and 2014, were retrieved and eight were selected for inclusion in this analysis. In total, 3967 patients were analyzed in these eight studies, three studies were single-center studies, and three included fewer than 200 patients. Two studies did not consider any surgical characteristic for analysis; source control adequacy was evaluated to some extent in six but details such as source control timing were only assessed in two. Half of the studies did not consider antibiotic adequacy as a variable; and timing of antibiotic therapy was not assessed in any study. Five studies considered microbiological characteristics as potential factors that may impact outcome. Reported mortality included either ICU mortality or hospital mortality (three each), and 30-day and 4-month mortality. Risk factors associated with mortality could be categorized into three major classes: (1) patient comorbidity, (2) treatment, and (3) severity of illness. All studies reported severity of illness (variable expressed, either scoring systems, organ dysfunction, or need for organ support) as a risk factor for outcome; Acute Kidney Injury (AKI) was cited specifically in five studies. Patient characteristics included age (three studies), gender (one study), and infection source (two studies). Comorbidities that were associated with outcome included cirrhosis (three studies) and hematological cancer (one study). Lack of source control was reported as independently associated with poor outcome in three studies; inadequate antibiotic therapy in one.

## Which treatment duration is adequate for critically ill patients with complicated intra-abdominal infection? Which are the major determinants of antibiotic choice in patients with complicated intra-abdominal infection (cIAI)?

**Statement 41**

**If the patient is critically ill the treatment duration can be deferred until after a multi-disciplinary careful evaluation (Recommendation 1B).**

Inadequate source control and inappropriate antibiotics are key determinants of mortality in patients having intra-abdominal sepsis and associated bacteremia.

In order to describe characteristics of critically ill patients with secondary blood stream infection (BSI) of intra-abdominal origin and identify predictors of mortality, a retrospective, single-center study that evaluated patients admitted between January 2005 and January 2011 was recently published [67]. Logistic regression analysis revealed inadequate source control (*P* = 0.002) and inappropriate antibiotics (*P* = 0.016) to be independently associated with mortality. In non-critically ill patients with adequate source control procedure after cIAI, the duration of the antimicrobial therapy was well defined [6]. However, for critically ill patients, an individualized approach is always mandatory. Appropriate antibiotic therapy, is a cornerstone for the success.

**Statement 42**

**Principal determinants of antibiotic choice in critically ill patients are based on three parameters: 1) Severity of illness, 2) local ecology, and 3) risk factors of the host. Previous antibiotic use is associated with a higher development of multidrug resistant organisms (MDROs). Broad-spectrum antibiotic therapy, including combination of different antibiotic classes should be recommended in patients with septic shock, settings with high rates of MDRO, and previous antibiotic administration (Recommendation 1B).**

Abdominal sepsis is a common indication for admission to the ICU. In 2014, the EPIC II study [226], including 13,796 adult patients from 1265 ICUs in 75 countries, demonstrated ICU mortality was higher in patients with abdominal infections compared to those with other infections (29.4 vs. 24.4%, *P* < 0.001). In patients with septic shock, early appropriate empiric antimicrobial therapy has a significant impact on the outcome, independent of the site of infection [227]. Prompt institution of antimicrobial therapy that is active against the causative pathogen(s) is crucial in the treatment of patients with severe infection and sepsis. In fact, the Surviving Sepsis Campaign strongly recommends initiating antibiotic therapy within the first hour of recognition of severe sepsis, after suitable cultures have been obtained. In a retrospective analysis of a large dataset collected prospectively for the Surviving Sepsis Campaign, Ferrer et al. [228] demonstrated that delay in first antibiotic administration was associated with increased in-hospital mortality in patients with severe sepsis and septic shock.

An antimicrobial policy of de-escalation therapy consisting of the initial use of wide-spectrum antimicrobials followed by a reassessment of treatment when culture results are available should be a principle of antimicrobial stewardship in critically ill patients [229]. Studies have reported conflicting effects on outcomes with de-escalation in various groups of critically ill patients.

To assess the safety and the impact on in-hospital and 90-day mortality of antibiotic de-escalation in patients admitted to the ICU with severe sepsis or septic shock, a prospective study was published in 2014 [230].

By multivariate analysis, factors independently associated with in-hospital mortality were septic shock, elevated SOFA score the day of culture results, and inadequate empirical antimicrobial therapy. In contrast, de-escalation therapy was a protective factor. However, in 2014, a multicenter randomized trial investigating a strategy based on de-escalation of antibiotics resulted in prolonged duration of ICU stay but did not affect the mortality rate [231].

Recently, Montravers et al. demonstrated that de-escalation is a feasible option in patients with polymicrobial infections such as healthcare-associated IAIs [128]. In a context of a dedicated “antibiotic stewardship” program, de-escalation should be encouraged, whenever possible, to optimize antibiotic use [232].

Emphasis on MDRO epidemiology is needed to better understand current strategies of prevention and management of critically ill patients in ICUs [233].

A rational use of antibiotics is important in order to prevent the emergence of multidrug-resistant bacteria, especially in ICUs. In critically ill patients, positive cultures may actually represent contamination. Antibiotic stewardship for critically ill patients may be translated into the implementation of specific guidelines, which were largely promoted by the Surviving Sepsis Campaign, targeted to optimizing choice, dosage, and duration of antibiotics in order to improve outcomes and reduce the development of resistance [234, 235].

## What is the best management of patients with abdominal sepsis?

**Statement 43**

**Early identification of sepsis and prompt administration of intravenous fluids and vasopressors are always mandatory. Restoring a mean systemic arterial pressure of 65 to 70 mm Hg is a good initial goal during the hemodynamic support of patients with sepsis (Recommendation 1 A).**

The definition of sepsis (Sepsis-3) [19] has returned to the traditional views that sepsis is characterized by organ dysfunction attributed to an infection. Patients with at least two of three clinical abnormalities including Glasgow coma score of 14 or less, systolic blood pressure of 100 mmHg or less, and respiratory rate 22/min or greater may have poor outcome typical of sepsis. Importantly, qSOFA does not define sepsis but provides simple bedside criteria to screen adult patients with suspected infection. Sepsis is now defined as a life-threatening organ dysfunction caused by a dysregulated host response to infection. It can be clinically represented by an increase in the SOFA score of 2 points or more [19]. Norepinephrine is now the first-line vasopressor agent which is used to correct hypotension in the event of septic shock. It is more efficacious than dopamine and is more effective for reversing hypotension in patients with septic shock [71, 236–238]. Septic shock is defined as a subset of sepsis in which particularly profound circulatory, cellular, and metabolic abnormalities who are associated with higher risk of mortality than with sepsis alone. Patients with septic shock can be clinically identified by requirement for vasopressors to maintain a mean arterial pressure of 65 mmHg or greater and serum lactate level less than 2 mmol/L (>18 mg/dL) in the absence of hypovolemia [19]. Under this terminology, “severe sepsis” becomes superfluous. Sepsis should generally warrant greater levels of monitoring and interventions.

In patients with sepsis, the 2016 SCC guidelines suggest that initial hemodynamic resuscitation should be achieved within 3 h [71].

Fluid therapy is needed to improve microvascular blood flow through an increased cardiac output as an essential part of the treatment of sepsis. A fluid challenge should incorporate four determinant elements [236]: (1) crystalloid solutions should be the first choice because they are well tolerated and cheap; (2) fluids should be infused rapidly to induce a quick response but not so fast that an artificial stress response develops; (3) the goal should be an increase in systemic arterial pressure; and (4) avoidance of pulmonary edema which is the most serious complication of fluid infusion through appropriate monitoring that is necessary to prevent edema occurrence.

Hypotension is the most common indicator of inadequate perfusion. Restoring a mean arterial pressure of 65 to 70 mmHg is a good initial goal during hemodynamic support of patients with sepsis [239].

**Statement 44**

**Fluid overload should be avoided in patients with generalized peritonitis, (Recommendation 1C).**

In patients with generalized peritonitis, fluid resuscitation should avoid fluid overload, which may aggravate gut edema and lead to increased intra-abdominal pressure (IAP) [239].

The systemic inflammatory response syndrome, increased vascular permeability, and aggressive crystalloid resuscitation predispose to fluid sequestration and collection in the peritoneum. Patients with advanced sepsis commonly develop bowel edema. These changes along with an associated forced closure of the abdominal wall may result in increased IAP ultimately leading to intra-abdominal hypertension (IAH) [238]. Elevated IAP may reduce both regional and global perfusion resulting in significant organ failure. An uncontrolled IAH, with an IAP exceeding 20 mmHg, and new organ failure onset leads to abdominal compartment syndrome (ACS). ACS is a potentially lethal complication affecting splanchnic, cardiovascular, pulmonary, renal, and central nervous systems [117, 240].

## What are the role of the adjunctive therapies in sepsis?

**Statement 45**

**There is currently insufficient evidence supporting the use of any adjunctive therapy in patients with septic shock due to intra-abdominal infection (No Recommendation).**

Although source control, antimicrobial therapy, and supportive therapies remain the cornerstone of treatment, especially in the early phase of sepsis, the identification of adjunctive therapies may play an important role in patients with ongoing sepsis.

In septic patients, mortality is higher when both pro- and anti-inflammatory cytokine levels are high [241, 242]. The rationale of using extracorporeal blood purification in patients with septic shock is to modulate the immune response. Blood purification for sepsis has consisted of various techniques: high-volume hemofiltration, high-adsorption hemofiltration, high cut-off membrane hemofiltration, plasma exchange, and hybrid systems [243].

A systematic review and meta-analysis of randomized trials to determine the association between various blood purification techniques and all-cause mortality in humans with sepsis was published in 2013 [244]. Ten single-center and six multicenter studies were identified. Ten trials reported patients with either severe sepsis or septic shock, while five trials reported only patients with a diagnosis of sepsis. One trial included patients with sepsis, severe sepsis, or septic shock. The blood purification techniques used included hemoperfusion (ten trials), hemofiltration (four trials), and plasma exchange (two trials).

Overall, blood purification decreased mortality compared with no blood purification. However, these results were driven mainly by hemoperfusion. Pooling of all trials of blood purification for treatment of sepsis was no longer associated with lower mortality after excluding trials using polymyxin B hemoperfusion. PMB-HP has been debated in recent years [245-246]. PMB-HP represents a promising strategy, but the Franch, prospective, multicenter, randomized controlled trial (ABDOMIX group) enrolling 243 patients with septic shock within 12 hours after emergency surgery for peritonitis related to organ perforation [247] failed to demonstrate a benefit. More recently the EUPRATHES (evaluating Polymyxin B Hemoperfusion in a randomized controlled study of adults treated for endotoxemia and septic shock) trial of PMB-HP in patients with septic shock and confirmed endotoxemia also failed to show an improved survival [248].

The use of intravenous immunoglobulin for treating patients with surgical sepsis is controversial. It is based on a potential benefit related to neutralization of endotoxin and various bacterial products. Intravenous immunoglobulin provides antibodies that can neutralize circulating exotoxins produced by organisms and may modulate the systemic inflammatory response induced by cytokine stimulation [249].

In order to evaluate the effects of intravenous immunoglobulin (IVIG) as adjunctive therapy in patients with bacterial sepsis or septic shock on mortality, bacteriological failure rates, and duration of stay in hospital, a Cochrane review was published in 2013 [250]. Forty-three RCTs comparing IVIG (monoclonal or polyclonal) with placebo or no intervention in patients of any age with bacterial sepsis or septic shock were reviewed. Subgroup analysis of ten polyclonal IVIG trials (*n* = 1430) and seven trials on IgM-enriched polyclonal IVIG (*n* = 528) showed significant reductions in mortality in adults with sepsis compared to placebo or no intervention. Pooled analysis of polyclonal and monoclonal IVIG was not done due to clinical heterogeneity. Polyclonal IVIG reduced mortality among adults with sepsis however this benefit was not seen in trials with low risk of bias.

A review of the mechanisms of action and meta-analysis of the clinical effectiveness was recently published [251]. The meta-analysis of 18 RCTs indicated that the use of IVIG reduces the mortality risk of septic patients. Low study quality, heterogeneous dosing regimens and type of Ig preparations, and different control interventions (placebo or albumin) are thought to have probably influenced results. Thus, the study showed that the use of IVIG therapy in adult septic patients may have a rationale and seems to be associated with a reduced mortality. However, the available evidence is not clearly sufficient to support the widespread use of IVIG in the treatment of sepsis.

A meta-analysis of randomized controlled studies published in 2007 compared IVIG preparations [252]. The meta-analysis demonstrated a strong trend in favor of an immunoglobulin preparation enriched with IgA and IgM (IgGAM) compared with preparations containing only IgG. 2016 Surviving Sepsis Campaign guidelines do not support the use of IVIG therapy [71].

## Which inflammatory mediators are involved in intra-abdominal sepsis? Are they useful markers in clinical practice?

**Statement 46**

**Inflammatory biomarkers require further controlled studies before their measurement guides clinical care of critically ill/injured patients (No recommendation).**

Inflammatory and protein mediators (cytokine, chemokine, acute phase proteins) play an important, but still not completely understood, role in the morbidity and mortality of abdominal sepsis.

Their potential utility includes function as (1) diagnostic/prognostic biomarkers, (2) therapeutic targets, and (3) elucidation of the pathogenic mechanisms of sepsis or injury-related organ dysfunction.

However, there is no consensus on the clinical use of mediators in diagnosing or managing intra-abdominal sepsis or injury. A MEDLINE, PubMed, EMBASE, and the Cochrane Library review on inflammatory mediators in abdominal sepsis was published in 2015 [253].

Serum PCT and C-reactive protein (CRP) appear to be useful to rule out infection or monitor therapy. There have been 33 studies exploring CRP as a marker for abdominal infection or complications after surgery. Kinetic studies demonstrated CRP levels elevate on post-operative day (POD) 1, peak from POD2 to POD3, and decline by POD5 provided that there is no complication or infection. Four reports suggest a persistent threshold of greater than 100 mg/L might indicate abscess/septic complications [254–257]; other studies have refuted this conclusion, leaving uncertainty for clinical utility [258–262].

Twelve trials including two randomized controlled studies evaluated the role of PCT as an indicator to diagnose infection, predict outcomes, or guide treatment of abdominal sepsis.

In some studies, persistent high levels of PCT were associated with infection and or increased septic mortality in patients with sepsis [263, 264], while other studies have not confirmed PCT as an accurate marker for sepsis or to predict a patient’s response to the initial treatment [201, 265].

The role of IL-6 as a marker to diagnose sepsis or predict outcomes has also been studied. However, its role remains uncertain and a wide range of cut-off values had been used (from 12 to 2760 pg/mL). Some studies considered IL-6 an indicator for sepsis or for predicting outcome and mortality [266–268]. However, other studies do not support the use of IL-6 as a valid sepsis biomarker [269, 270].

Recent studies have reported endogenous DAMPs (mtDNA, HMGB1) released as a consequence of tissue injury or infection to be promising biomarkers [271, 272]; however, the evidence supporting their role is still limited and their use undefined.

**Statement 47**

**Consideration should be given to draining ascites in the critically ill patient treated for peritonitis, especially if the ascites is associated with IAH (Recommendation 1C).**

It has been found that increased levels of both systemic and peritoneal cytokines are associated with post-operative complications, which may discriminate survivors from those who died [273, 274].

These data suggest that measurement of peritoneal cytokines could be a potentially important method to determine and follow the patient’s inflammatory reaction.

Although data from research with animal models [275] having inflammatory bowel disease [265, 276] are suggestive, direct evidence does not yet exist to prove that effectively draining this peritoneal fluid makes a difference to complications or survival.

Severe IAH has been shown to significantly reduce perfusion to the intestinal mucosa, which ultimately increases intestinal permeability and results in systemic endotoxemia aggravating the sepsis cascade. Damaged gut is a continual source of inflammation and MODS, referred to as the “Motor of MSOF” [277–282], by inducing the production of cytokines and other biomediators. The release of endotoxin induces production of cytokines, including interleukin (IL)-6, IL-1B, IL-8, tumor necrosis factor-α (TNF-α), and other mediators and movement of these mediators into the systemic circulation may be largely modulated by the mesenteric lymphatic channels, as demonstrated in animal models [283].

In clinical practice, the accumulation of intraperitoneal mediators can be removed by either percutaneous drainage and negative pressure therapy with an open abdomen. Percutaneous drainage is recommended to treat IAH if it is possible to do safely, as it may obviate the need for decompressive laparotomy [119, 284]. If percutaneous drainage is not safely possible, NPPT may be another appropriate option.

A single-center RCT published in 2015 conducted on critically ill and injured patients with an average APACHE score above 22 in critically ill and an ISS over 23 in the injured compared commercial NPPT system with the Barkers VAC PAC in a randomized fashion [125]. Although this study did not find a difference in actual peritoneal fluid drainage or in the behavior of the high level mediators examined (IL-1β, IL-8, IL-10, or IL-12, p70, or TNFα), there was a survival difference in favor of the commercial system. However, it is possible that patient heterogeneity in the complex setting of mixed critical care populations can alone explain the findings, and thus, further studies are required.

## Conclusions

In the Appendix, all the recommendations from the “Dublin WSES 2016 Intra-Abdominal Infections Consensus Conference” are listed.

## Appendix

### Classification, diagnosis, and risk factors

Statement 1

The term “intra-abdominal infections” describes a wide heterogeneity of clinical conditions. The anatomical extent of infection, the presumed pathogens involved, risk factors for major resistance patterns, and the patient's clinical condition should be assessed independently so as to classify patients (*Recommendation 1 C*).

Statement 2

Early clinical evaluation is essential for diagnosing IAIs. It helps to optimize diagnostic testing and can result in earlier implementation of a proper management plan (Recommendation 1C).

Statement 3

A step-up approach for diagnosis should be used and tailored to the clinical setting, resources, patient’s age beginning with clinical and laboratory examination and progressing to imaging examinations (Recommendation 1C).

Statement 4

Patient factors are essential when addressing treatment outcome, as advanced age, associated comorbidity, pre-existing disease, and physiologic status greatly influence outcomes (e.g., mortality) (Recommendation 2 C).

Statement 5

Disease factors are essential to consider when addressing risk for treatment failure. (Recommendation 2 C).

Statement 6

While age alone is not decisive for outcome it should be recognized that patients with an accumulated number of risk factors, including advanced age, high disease severity and presenting in sepsis or septic shock, have a very high risk of death. Palliative care should be actively discussed when conditions indicate that operative treatment is futile (Recommendation 2 C).

Statement 7

Prognostic scoring systems for complicated IAIs may be useful in clinical practise, especially for audit and research. Scoring systems can be broadly divided into two groups: general organ failure severity (ICU) scores and peritonitis-specific (Surgical) scores. The Sequential Organ Failure Assessment (SOFA) score allows physicians to follow the evolving disease process in critically ill patients in ICU (Recommendation 2 A).

However, although these scores may help guide clinical care, their use in individual patients is much less predictive.

### Source control

Statement 8

Most patients with cIAIs and sepsis/septic shock should undergo an urgent source control procedure; source control can be delayed in less severely-ill patients when the circumstances are appropriate (Recommendation 2 C).

Statement 9

Highly selected patients with perforated diverticulitis (including those with an abscess <4 cm in diameter), a peri-appendiceal mass, or a perforated peptic ulcer can be managed without definitive source control if responding satisfactorily to antimicrobial therapy and other supportive measures. (Recommendation 1 B).

Statement 10

Laparoscopic appendectomy represents the first choice for most patients with acute appendicitis where appropriate resources and skills are available (Recommendation 1 A).

Statement 11

There is no evidence of any significant advantages between laparoscopic and open repair of perforated peptic ulcer (PPU). However, laparoscopy has less post-operative pain and shorter hospital stay (Recommendation 1 A).

Statement 12

Early laparoscopic cholecystectomy is safe and feasible in acute cholecystitis and should be the preferred choice in absence of contraindications to pneumoperitoneum, even in high risk patients, where appropriate resources and skills are available (Recommendation 1A).

Statement 13

Laparoscopic lavage is not recommended in Hinchey IV diverticulitis because it can not achieve adequate source control. Laparoscopic lavage is safe and not inferior to sigmoid resection in case of Hinchey III but it is not considered the preferred choice, given the lack of evidence of major benefits (Recommendation 1A).

Statement 14

Laparoscopic sigmoid resection is feasible and safe in selected patients, hemodynamically stable, without significant comorbidities and with onset of peritonitis <12-24 hours, only if specific advanced laparoscopic colorectal expertise is available (Recommendation 2 C).

Statement 15

Planned relaparotomy is not recommended as a general strategy in patients with secondary peritonitis (Recommendation 1A).

Statement 16

There is insufficient evidence to advocate damage control surgery as general strategy in patients with secondary peritonitis (Recommendation 1C).

Statement 17

Damage control surgery may be an option in selected significantly physiologically deranged patients with ongoing sepsis (Recommendation 2C).

Statement 18

Temporary abdominal closure using negative pressure therapy (NPT) can be useful to decrease the time to definitive abdominal closure (Recommendation 1B). Prolonged NPT may increase the risk of enteric fistulae.

### Antimicrobial therapy

Statement 19

Intraperitoneal specimens for microbiological evaluation from the site of infection are always recommended for patients with HA-IAIs or with CA-IAIs at risk for resistant pathogens (previous antimicrobial therapy) and in critically ill patients (Recommendation 1 B).

Statement 20

Intraperitoneal specimens should be collected in every re-operation (Recommendation 1 C).

Statement 21

Appropriate intraperitoneal specimen is peritoneal fluid/tissue collected from the site of infection *(Recommendation 1 C).*

Statement 22

Sufficient abdominal fluid/tissue volume (usually at least 1–2 mL of fluid) should be collected and transported to the microbiology laboratory using a transport system that properly handles and *preserves the samples to avoid damage or compromise their integrity (Recommendation 1 C).*

Statement 23

At the laboratory the intraperitoneal specimen should undergo Gram stain, aerobic and anaerobic culture, and antibiotic susceptibility testing (Recommendation 1 C).

Statement 24

Empirical antimicrobial therapy should be based on local epidemiology, individual patient risk factors for difficult to treat pathogens, clinical severity of infection, and infection source (Recommendation 1 C).

Statement 25

The correct dose and correct administration of antimicrobials should include: 1) loading dose when indicated, especially in critically ill patients; 2) extended or prolonged infusion for beta-lactam antibiotics; 3) peritoneal distribution (Recommendation 2 C).

Statement 26

The patient should be reassessed when the results of microbiological testing are available. Antimicrobial de-escalation or withdrawal should be considered (Recommendation 1C).

Statement 27

In the settings with a high incidence of ESBL-producing Enterobacteriaceae, the extended use of cephalosporins should be discouraged and should be limited to pathogen-directed therapy because of its selective pressure resulting in emergence of resistance (Recommendation 1C).

Statement 28

Extended use of fluoroquinolones (FQ) should be discouraged because of its selective pressure (mainly ESBLs producing Entrobacteriaceae and MRSA). They should be generally used in patients with allergy to beta-lactams (Recommendation 1C).

Statement 29

For patients with CA-IAIs, agents with a narrower spectrum of activity should be suggested. However, according to local ecology anti-ESBL-producer coverage may be warranted. By contrast, for patients with HA-IAIs, antimicrobial regimens with broader spectra of activity are preferred (Recommendation 1B).

Statement 30

Carbapenem sparing treatment should be recommended particularly in the settings where there is a high incidence of carbapenem resistant K. pneumoniae (Recommendation 1B).

Statement 31

Antimicrobial resistance among enterococcal isolates (ampicillin, gentamcin or vancomycin resistance) is mostly found in nosocomial (postoperative or tertiary) peritonitis. In Vancomycin-resistant Enterococcus (VRE), treatment with linezolid (monomicrobial infection) or tigecycline (polymicrobial infection) is appropriate (Recommendation 1 B).

Statement 32

The presence of Candida spp. in the peritoneal samples is a factor of poor prognosis (Recommendation 1C).

Statement 33

Two situations justify an empirical antifungal therapy: patients with septic shock in community-acquired infections or patients with post-operative infections (Recommendation 2 C).

Statement 34

In the setting of uncomplicated acute cholecystitis and acute appendicitis post-operative antimicrobial therapy is not necessary (Recommendation 1A).

Statement 35

In patients with IAIs, when patients are not severely ill and when source control is complete, a short course (3–5 days) of post-operative therapy is suggested (Recommendation 1A).

Statement 36

In patients with ongoing or persistent IAIs, the decision to continue, revise, or stop antimicrobial therapy should be made on the basis of clinician judgment and laboratory information (Recommendation 1 A).

Statement 37

Multifaceted interventions are more likely to improve antibiotic prescribing practices than simple, passive interventions. Didactic educational programs alone are generally ineffective.

(Recommendation 1B).

Statement 38

As a single intervention, implementation of locally adapted, interdisciplinary evidence based guidelines that incorporate risk stratification (severity and CA-IAIs versus HA-IAIs) and local resistance data most consistently improves components of AB prescribing for IAI (Recommendation 1 B).

Statement 39

Computer-assisted order entry, non-physician health care provider facilitation, and therapeutic drug monitoring can improve components of antibiotic prescribing practices, if resources are available (Recommendation 2 B).

### Critically ill patients

Statement 40

The lack of source control and antibiotic adequacy are the only modifiable risk factors for mortality in patients with cIAIs admitted to the ICU. Organ dysfunction is associated with worse outcomes (Recommendation 1 B).

Statement 41

If the patient is critically ill the treatment duration can be deferred until after a multi-disciplinary careful evaluation (Recommendation 1B).

Statement 42

Principal determinants of antibiotic choice in critically ill patients are based on three parameters: 1) Severity of illness, 2) local ecology, and 3) risk factors of the host. Previous antibiotic use is associated with a higher development of multidrug resistant organisms (MDROs). Broad-spectrum antibiotic therapy, including combination of different antibiotic classes should be recommended in patients with septic shock, settings with high rates of MDRO, and previous antibiotic administration (Recommendation 1B).

Statement 43

Early identification of sepsis and prompt administration of intravenous fluids and vasopressors are always mandatory. Restoring a mean systemic arterial pressure of 65 to 70 mm Hg is a good initial goal during the hemodynamic support of patients with sepsis (Recommendation 1 A).

Statement 44

Fluid overload should be avoided in patients with generalized peritonitis, (Recommendation 1C).

Statement 45

There is currently insufficient evidence supporting the use of any adjunctive therapy in patients with septic shock due to intra-abdominal infection (No Recommendation).

Statement 46

Inflammatory biomarkers require further controlled studies before their measurement guides clinical care of critically ill/injured patients (No recommendation).

Statement 47

Consideration should be given to draining ascites in the critically ill patient treated for peritonitis, especially if the ascites is associated with IAH (Recommendation 1C).

## Abbreviations

CA-IAIs: Community-acquired intra-abdominal infections
HA-IAIs: Hospital-acquired intra-abdominal infections
HCAI: Healthcare-associated infection
IAIs: Intra-abdominal infections
MDROs: Multidrug-resistant organisms
